# Transcriptional and metabolomic responses of *Methylococcus capsulatus* Bath to nitrogen source and temperature downshift

**DOI:** 10.3389/fmicb.2023.1259015

**Published:** 2023-10-20

**Authors:** Ashwini Ashok Bedekar, Anshu Deewan, Sujit S. Jagtap, David A. Parker, Ping Liu, Roderick I. Mackie, Christopher V. Rao

**Affiliations:** ^1^Energy and Biosciences Institute, Materials Research Laboratory, University of Illinois at Urbana-Champaign, Champaign, IL, United States; ^2^Carl R. Woese Institute for Genomic Biology, University of Illinois at Urbana-Champaign, Champaign, IL, United States; ^3^Department of Chemical and Biomolecular Engineering, University of Illinois at Urbana-Champaign, Champaign, IL, United States; ^4^Shell Exploration and Production Inc., Westhollow Technology Center, Houston, TX, United States; ^5^Department of Animal Sciences, University of Illinois at Urbana-Champaign, Champaign, IL, United States

**Keywords:** *Methylococcus capsulatus Bath*, methane metabolism, nitrogen metabolism, transcriptomics, metabolomics

## Abstract

Methanotrophs play a significant role in methane oxidation, because they are the only biological methane sink present in nature. The methane monooxygenase enzyme oxidizes methane or ammonia into methanol or hydroxylamine, respectively. While much is known about central carbon metabolism in methanotrophs, far less is known about nitrogen metabolism. In this study, we investigated how *Methylococcus capsulatus* Bath, a methane-oxidizing bacterium, responds to nitrogen source and temperature. Batch culture experiments were conducted using nitrate or ammonium as nitrogen sources at both 37°C and 42°C. While growth rates with nitrate and ammonium were comparable at 42°C, a significant growth advantage was observed with ammonium at 37°C. Utilization of nitrate was higher at 42°C than at 37°C, especially in the first 24 h. Use of ammonium remained constant between 42°C and 37°C; however, nitrite buildup and conversion to ammonia were found to be temperature-dependent processes. We performed RNA-seq to understand the underlying molecular mechanisms, and the results revealed complex transcriptional changes in response to varying conditions. Different gene expression patterns connected to respiration, nitrate and ammonia metabolism, methane oxidation, and amino acid biosynthesis were identified using gene ontology analysis. Notably, key pathways with variable expression profiles included oxidative phosphorylation and methane and methanol oxidation. Additionally, there were transcription levels that varied for genes related to nitrogen metabolism, particularly for ammonia oxidation, nitrate reduction, and transporters. Quantitative PCR was used to validate these transcriptional changes. Analyses of intracellular metabolites revealed changes in fatty acids, amino acids, central carbon intermediates, and nitrogen bases in response to various nitrogen sources and temperatures. Overall, our results offer improved understanding of the intricate interactions between nitrogen availability, temperature, and gene expression in *M. capsulatus* Bath. This study enhances our understanding of microbial adaptation strategies, offering potential applications in biotechnological and environmental contexts.

## 1. Introduction

Methanotrophs are bacteria capable of utilizing methane as their sole carbon and energy source (Patt et al., 1974; Sahoo et al., 2021). These methane-oxidizing bacteria act as a biological methane sink for the potent greenhouse gas methane (Patel et al., 2022). Some of methanotrophs are aerobic, whereas others are anaerobic (Hanson and Hanson, 1996; Strong et al., 2016; Case et al., 2017; Su et al., 2017; Steinsdóttir et al., 2022). In the case of aerobic methanotrophs, the first step in methane metabolism involves the oxidation of methane to methanol by enzymes known as methane monooxygenases (Lawton and Rosenzweig, 2016; Patel et al., 2023). The next step involves the oxidation of methanol to formaldehyde by methanol dehydrogenase. Formaldehyde can further be oxidized to CO_2_ or enter central metabolism via the ribulose monophosphate pathway or serine cycle depending on the type of methanotroph (Theisen and Murrell, 2005; Haynes and Gonzalez, 2014; Wang et al., 2014; Koo and Rosenzweig, 2021; Hu et al., 2022; Webster et al., 2022). Type I methanotrophs from gammaproteobacteria use the ribulose monophosphate pathway and type II methanotrophs from alphaproteobacteria use the serine cycle for formaldehyde assimilation (Whittenbury and Dalton, 1981; Hakobyan and Liesack, 2020).

Nitrogen sources can influence the growth rate and other physiological aspects of methanotrophs (Sugden et al., 2021). Most methanotrophs can utilize either nitrate or ammonium as a nitrogen source (Nyerges et al., 2010; Tays et al., 2018), and some can also fix atmospheric nitrogen (Auman et al., 2001; Li et al., 2021). As ammonium is the least expensive in terms of energy, it can be immediately assimilated into cell biomass. Early research on methanotrophs showed that methane monooxygenases could oxidize ammonium by competitively blocking methane oxidation as methane and ammonium have similar structures (Bédard and Knowles, 1989; Holmes et al., 1995; Bodelier and Laanbroek, 2004). The comparison of the growth of methanotrophs on nitrate and ammonium has shown that inhibition of methane oxidation by ammonium and the toxicity of ammonium oxidation by-products vary among methanotrophs (Nyerges and Stein, 2009; Tays et al., 2018). While extensive research has been conducted on the role of both particulate methane monooxygenases (pMMO) and soluble methane monoxygenases (sMMO) in methane oxidation, studies differentiating their involvement in ammonia conversion are relatively limited. Ammonium oxidation by pMMO might be easier than being transported to the cytoplasm for sMMO to function (Ayub et al., 2022). While nitrate addition might stimulate both types of methanotrophs in paddy soil, ammonium addition only enriched type I methanotrophs while suppressing the growth of type II methanotrophs (Hu and Lu, 2015). While the methanotrophic strains lacking hydroxylamine oxidoreductase (HAO) are poor nitrifiers, the HAO-expressing strains may effectively oxidize ammonia (Nyerges and Stein, 2009; Stein and Klotz, 2011; Choi et al., 2023). Methane monooxygenases or ammonia monooxygenases linked to membranes oxidize ammonium in strains carrying the HAO gene, producing the intermediate hydroxylamine (Mohammadi et al., 2017). By means of hydroxylamine oxidoreductase, the cytotoxic hydroxylamine is converted into nitrite (Poret-Peterson et al., 2008). Nitrite is further oxidized to form nitric oxide, which is then reduced by nitric oxide reductase to form nitrous oxide.

Various environmental factors such as pH, temperature, moisture content, and nutrient content affect the microbial methane oxidation process (Bender and Conrad, 1995; Park et al., 2005; Webster et al., 2022). Among these factors, temperature affects methane diffusion and regulates the oxidative activity of methanotrophs (Visvanathan et al., 1999; Scheutz and Kjeldsen, 2004). A change in methanotrophic population was observed when the forest soil and rice field soil were incubated at different temperatures at elevated methane concentrations (Mohanty et al., 2007). Few studies have shown the effect of temperature on pure methanotrophic cultures and products (Soni et al., 1998; Pérez et al., 2019). Several studies on methanotrophs have discussed the strain-specific preferences of methanotrophs when provided with different carbon–nitrogen source combinations and their effect on metabolite production (Nyerges et al., 2010; Tays et al., 2018; Lazic et al., 2021).

*M. capsulatus* Bath is a model methanotroph that has been widely studied in various research areas (Christoffersen et al., 2015; Ishikawa et al., 2018; Jawaharraj et al., 2021; Lu et al., 2023). Attempts have been made to genetically engineer this bacteria for the production of value-added chemicals such as mevalonate (Jeong et al., 2023). This methanotroph has been commercially used for single-cell protein production from synthetic nitrogen and natural gas (Bajić et al., 2022). Additionally, industries such as Norferm Danmark A/S reported use of *M. capsulatus* Bath for the production of single cell protein, BioProtein (Bothe et al., 2002). However, efforts to optimize the culturing conditions for this bacterium are still ongoing (Jang et al., 2023).

In this work, we investigated the growth responses of *M. capsulatus* Bath in a series of batch culture experiments when exposed to nitrate or ammonium as nitrogen sources at temperatures of 37°C and 42°C. Most interestingly, nitrate usage was temperature-dependent while the use of ammonia was similar over the studied temperature ranges. To better characterize the response to nitrogen source and temperature, we performed transcriptomics using RNA-seq and metabolomics using gas chromatography–mass spectrometry (GC–MS). The accuracy of transcriptional changes was confirmed by quantitative PCR. This study provides a more detailed understanding of how *M. capsulatus* Bath adapts to nitrogen availability and temperature fluctuations. These insights into microbial adaptations offer novel approaches for methane utilization.

## 2. Materials and methods

### 2.1. Strains, media, and growth conditions

The *M. capsulatus* Bath strain used in this study was obtained from the American Type Culture Collection (ATCC 33009). Cultures were grown using either nitrate mineral salt medium (NMS: 1% KNO_3_, 1% MgSO_4_·7H_2_O, 0.2% CaCl_2_·2H_2_O, 0.4 mg/L ferric EDTA and 500 μL/L Pfennig’s trace element solution) or ammonium mineral salt medium (AMS: 0.5% NH_4_Cl, 1% MgSO_4_·7H_2_O, 0.2% CaCl_2_·2H_2_O, 0.4 mg/L Ferric EDTA and 500 μL/L Pfennig’s trace element solution). Pfennig’s trace element solution contains 5% EDTA, 0.1% ZnSO_4_·7H_2_O, 0.03% MnCl_2_·4H_2_O, 0.03% H_3_BO_3_, 0.2% CoCl_2_·6H_2_O, 0.03% CuCl_2_·2H_2_O, 2.2% FeSO_4_·6H_2_O, 0.02% NiCl_2_·6H_2_O, 0.03% Na_2_MoO_4_·2H_2_O, and 0.01% Na_2_SeO_3_ (Lippert and Pfennig, 1969). Phosphate buffer was used to adjust the medium pH to 6.8. Wheaton serum bottles (250 mL) filled with 50 mL medium and sealed with butyl-rubber stoppers with aluminum vial crimp seals were used for all the experiments. Methane and air were provided in 1:1 ratio through a 0.22 μm filter-fitted syringe. Serum bottles were inoculated with a starting O.D. of 0.02 and incubated at 37°C or 42°C at 250 rpm. All experiments were performed in triplicate.

### 2.2. Experimental procedure for RNA-seq

Total RNA was extracted from cell pellets using the RNeasy plus mini kit from Qiagen with minor modifications (Larsen and Karlsen, 2016). *M. capsulatus* Bath cells (up to 1 × 10^9^) were collected after 15 h of growth and resuspended in 700 μL of buffer RLT (with previously added β-mercaptoethanol) from the RNeasy mini kit. Approximately 50 μL of acid washed glass beads (Sigma, acid washed, 150–600 μm) were added and the cell pellets were disrupted using a FastPrep-24 homogenizer at a speed of 5 m/s for 30 s twice with cooling on ice between beatings. The purification of the cell lysates was performed according to the protocol provided with the kit. Turbo RNase-free DNase kit from ThermoFisher was used for further treatment of extracted RNA as per protocol and purification was done using the RNeasy mini kit protocol (RNA clean up). The RNAseq libraries were prepared with Illumina’s ‘TruSeq Stranded mRNAseq Sample Prep kit’. The libraries were quantitated by qPCR and sequenced on one lane for 101 cycles from one end of the fragments on a NovaSeq 6,000 using a NovaSeq SP reagent kit. Fastq files were generated and demultiplexed with the bcl2fastq v2.20 Conversion Software (Illumina). The sequencing files were uploaded to NCBI (NCBI Bioproject Accession: PRJNA796612).

### 2.3. RNA-seq data analysis

Low-quality reads and adaptor sequences were trimmed using Trimmomatic (Bolger et al., 2014). The quality scores of trimmed reads were analyzed using FastQC (Andrews, 2010). Reads were mapped to the *M. capsulatus* Bath reference genome (NCBI Accession GCA_000008325.1) with the Burrows-Wheeler Alignment Tool version 0.7.17 (Li and Durbin, 2009). For each sample, 75–90% of the reads were successfully mapped to the genome. FeatureCounts from the Subread package, v2.0.0 was used to calculate read counts (Liu et al., 2020). Differential expression analysis was performed on the reads counts in R v4.1.1 using edgeR v4.1.1 and limma v3.34.1 (Robinson et al., 2010; Ritchie et al., 2015). Graphical representation of expression data was constructed using R packages: PCAtools v2.4.0, Glimma v2.0.0, and gplots v3.1.1 (Su et al., 2017). The data was row-wise normalized before plotting heatmaps (using the scale function in R), first by centering (subtracting the row mean from each value) and then scaling (dividing each data point by the row’s standard deviation). Heatmaps were plotted using gplots heatmap.2 function. Functional annotation of *M. capsulatus* Bath was obtained from NCBI (accession GCA_000008325.1). Data analysis scripts, along with the results, were uploaded at https://github.com/raogroupuiuc/MCbath-nitrogen-RNAseq.

Genes showing significantly differential transcript abundance (value of *p* <0.05) were further subcategorized based on their gene ontology terms (enriched genes for pathways) using the BioCyc pathway/genome database collection.

### 2.4. Quantitative PCR

Total mRNA was extracted using Qiagen’s RNeasy Mini kit as described above. Total mRNA (1 μg) was then treated with TURBO DNA-free DNase using Ambion’s TURBO DNA-free kit (Thermo Fisher, Carlsbad, CA) to remove genomic DNA. cDNA was synthesized from mRNA using Bio-Rad’s iScript cDNA synthesis kit. The qPCR experiments were carried out in a 384 well plate using a Roche LightCycler 480 system with the SsoAdvanced Universal SYBR Green Supermix kit (Bio-Rad, Hercules, CA). Primers were designed using the online PrimerQuest tool provided by Integrated DNA Technologies and are listed in Supplementary Table S1. The *rpoA* gene encoding the alpha subunit of RNA polymerase (MCA_11500) was used as a reference gene. All data points were collected from three biological replicates.

### 2.5. Sample preparation for metabolome analysis

For intracellular metabolite analysis, the fast filtration sampling method was used as previously described (Kim et al., 2013; Jagtap et al., 2019). Briefly, 10 mL of cells grown in NMS and AMS media at 37 and 42°C, cells were collected at 15 h time point and vacuum-filtered using a vacuum manifold system (Vac-Man Laboratory Vacuum Manifold, Promega, Madison, WI, US) assembled with a nylon membrane filter (0.45 μm pore size, 13 mm diameter, Whatman, Piscataway, NJ, US) and a sterile filter holder (Millipore, Billerica, MA, US). The filtered cells were then washed with 2.5 mL of distilled water at room temperature. The entire process was completed within 1 min. The filter membrane containing the washed cells was quickly mixed with 1 mL of the pre-chilled isopropanol:acetonitrile:water mixture (3,3:2, v/v). Cell samples were subsequently extracted by ultrasound (5 × 1 min) with the QSonica Microson XL2000 Ultrasonic Homogenizer (Qsinica, LLC., CT, United States) at 4°C, centrifuged at 15000 × g for 3 min 4°C, and supernatants were collected and evaporated under vacuum. The samples were prepared for GC/MS analysis as described in the analytical methods section.

### 2.6. Analytical methods

#### 2.6.1. Headspace gas analysis

Methane and oxygen concentrations in headspace were monitored with a gas chromatograph (GC-2014; Shimadzu Corporation, Kyoto, Japan) equipped with a thermal conductivity detector (GC-TCD) over 40 h of incubation. Supelco custom-packed column (Packing 80/100 Hayesep Q) was used to perform headspace gas analysis. The carrier gas (Ar) flow rate was 30 mL/min with the injector and detector temperature set at 110°C, and the column temperature at 75°C. The gas samples were injected manually using a gas tight syringe.

#### 2.6.2. Measurement of nitrate, nitrite, and ammonia concentration in the medium

To determine the concentration of nitrate, ammonia, and nitrite in the culture medium, samples were collected after 15 h, 24 h, and 40 h of incubation. The cells were centrifuged at 16000 × g for 5 min, and the supernatant was used to determine nitrate, nitrite, and ammonia concentrations. Nitrate and nitrite concentrations in the NMS medium and nitrite concentration in the AMS medium were analyzed using the Cayman nitrate/nitrite colorimetric assay kit (Cayman Chemical, Ann Arbor, MI, United States) according to the manufacturer’s instructions.

For colorimetric determination of ammonium concentration, the indophenol blue method was used (Mann, 1963; Ivančič and Degobbis, 1984). The intensity of indophenol blue pigments formed due to the reaction between phenol and hypochlorite in presence of ammonia was measured at 630 nm.

#### 2.6.3. Metabolite analysis

Prior to the metabolomic analysis, culture samples collected at the 15 h time point were centrifuged at 16000 × g for 10 min and the supernatant was passed through 0.22 μm polyethersulfone syringe filter. The samples were derivatized with 100 μL methoxyamine hydrochloride (40 mg ml^−1^ in pyridine) for 90 min at 50°C and then with 100 μL MSTFA at 50°C for 120 min. Twenty microliters of the internal standard (hentriacontanoic acid, 1 mg/mL) was added to each sample prior to derivatization. Samples were analyzed on a GC/MS system (Agilent Inc., Palo Alto, CA, United States) consisting of an Agilent 7,890 gas chromatograph, an Agilent 5,975 mass selective detector, and HP 7683B autosampler. Gas chromatography was performed on a ZB-5MS (60 m × 0.32 mm I.D. and 0.25 μm film thickness) capillary column (Phenomenex, CA, United States). The inlet and MS interface temperatures were 25°C, and the ion source temperature was adjusted to 230°C. An aliquot of 1 μL was injected with the split ratio of 10:1. The helium carrier gas was kept at a constant flow rate of 2.4 mL/min. The temperature program was: 5 min isothermal heating at 70°C, followed by an oven temperature increase of 5°C/min to 310°C and a final 10 min at 310°C. The mass spectrometer was operated in positive electron impact mode (EI) at 69.9 eV ionization energy in m/z 30–800 scan range.

All known artificial peaks were identified and removed. MS peaks were evaluated by AMDIS 2.71 (NIST, Gaithersburg, MD, United States) program and metabolites were identified by a custom-built library (484 unique metabolites). To allow comparison between samples, all data were normalized to the internal standard in each chromatogram and the cells dry weight. The dry cell weight was measured by filtration of 50 mL of the cell culture (OD = 1) using a 0.2 μm cellulose acetate filter (Sartorius 11,107-47-N), followed by drying at 80°C (Long and Antoniewicz, 2014).

## 3. Results

### 3.1. Growth profiles of *M. capsulatus* Bath on different nitrogen sources at different temperatures

**Figure 1:**
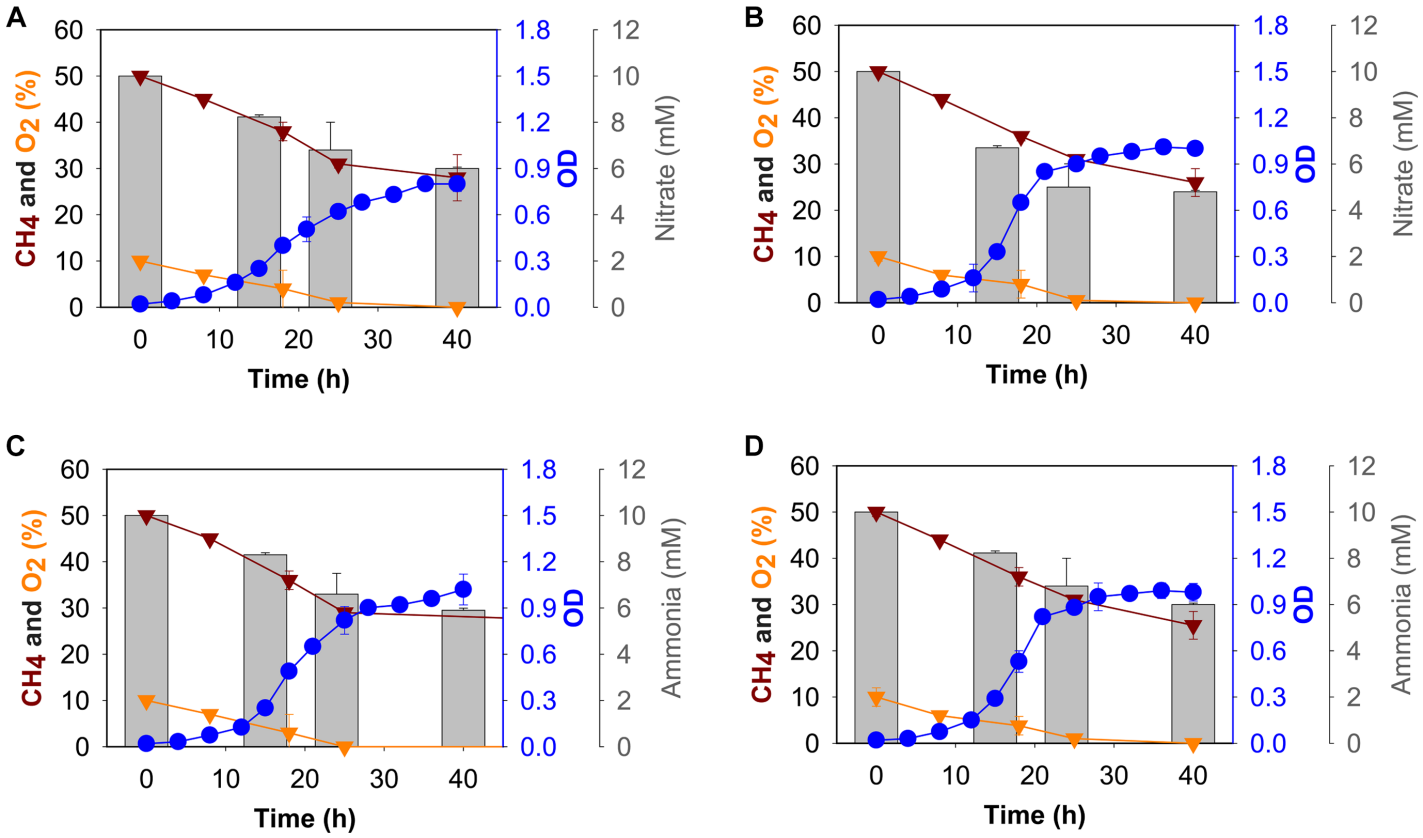
Growth (blue circles), gas (methane: dark red triangles, oxygen: gold triangles), and nitrogen source (grey histogram) consumption profiles of *M. capsulatus* Bath grown in nitrate mineral salt medium at 37°C **(A)**, 42°C **(B)**, and ammonium mineral salt medium at 37°C **(C)** and 42°C **(D)**.

To determine the effect of nitrogen source, we grew *M. capsulatus* Bath in batch culture using either nitrate (10 mM KNO_3_) or ammonium (10 mM NH_4_Cl) at 37°C or 42°C, and growth was measured over 40 h. All the cultures reached stationary phase after 24 h of growth (Figure 1) in tested conditions. The cultures were found to be oxygen limited after 24 h of growth, whereas methane was continually present in the gas headspace. We grew *M. capsulatus* Bath at 42°C and tested the effect of a temperature downshift at 37°C.

At 42°C, the growth rate of *M. capsulatus* Bath was 0.20 ± 0.03 h^−1^ and 0.20 ± 0.09 h^−1^ in nitrate and ammonium, respectively (Supplementary Table S2). The doubling time was 3 h and 47 min when nitrate was used and 3 h and 40 min when ammonium was used as a nitrogen source. At 37°C, growth rate was 0.17 ± 0.01 h^−1^ in nitrate and 0.19 ± 0.01 h^−1^ in ammonium, whereas a 4 h and 16 min doubling time was calculated when nitrate was used, and the doubling time was decreased to 3 h and 51 min when ammonium was used as a nitrogen source. Thus, no difference in growth was observed in nitrate and ammonium at 42°C, *M. capsulatus* Bath grew better at 37°C in ammonium as compared to nitrate, with a higher growth rate and a lower doubling time (Supplementary Table S2).

### 3.2. Nitrate/ammonia oxidation and nitrite production by *M. capsulatus* Bath

To determine nitrate and ammonia utilization by *M. capsulatus* Bath, we measured the nitrate/ammonia concentration in NMS/AMS medium, respectively. Samples were taken after 0 h, 15 h, 24 h, and 40 h of growth, the nitrate utilization rate was measured. *M. capsulatus* Bath was able to utilize more nitrate at 42°C as compared to 37°C when grown on NMS medium (Figures 1A,B). Nitrate utilization was higher during the first 24 h of fermentation (5 mM at 42°C and 4.2 mM at 37°C). After 48 h, *M. capsulatus* Bath was able to utilize 5.4 mM of nitrate when grown at 42°C and 4.7 mM of nitrate when grown at 37°C.

In methanotrophs, nitrate is reduced to nitrite by the nitrate reductase enzyme. We also tested the nitrite accumulation in *M. capsulatus* Bath growth medium (Supplementary Figure S1). At 42°C, 1.30 μM of nitrite was produced after 40 h of incubation, while at 37°C it was 0.71 uM. Nitrite is further reduced to ammonia by nitrite reductase. Ammonia was not detected in NMS medium at 42°C and 37°C.

In AMS medium, overall utilization of ammonia was comparatively similar at 42°C and 37°C (4 mM, and 4.1 mM respectively). Ammonia utilization decreased after 24 h of incubation (Figures 1C,D). Also, at 37°C, 0.1 μM of nitrite was produced after 40 h of incubation, while at 42°C, it was 0.14 μM (Supplementary Figure S1).

### 3.3. Transcriptional analysis of *M. capsulatus* Bath

**Figure 2:**
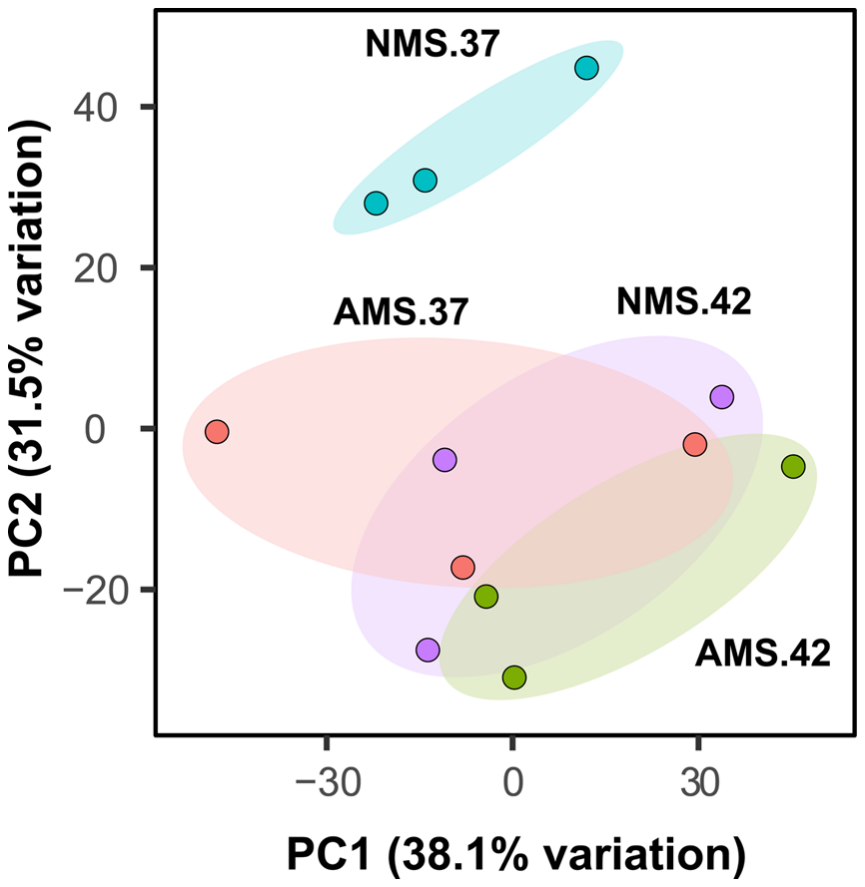
Principal component analysis plot generated from transcript abundance of *M. capsulatus* Bath grown on nitrate at 42°C (NMS 42), nitrate at 37°C (NMS 37), ammonium at 42°C (AMS 42), and ammonium at 37°C (AMS 37). Samples were collected in exponential growth phase at 15 h time point. RNA-seq data was collected in triplicate for each condition.

We hypothesized that gene expression profiles of *M. capsulatus* Bath would change in response to nitrogen source and temperature downshift. To test this hypothesis, analysis of transcript abundance was carried out in *M. capsulatus* Bath grown on either nitrate (NMS) or ammonia (AMS) at 37°C or 42°C. All experiments were performed using triplicate cultures. A total of 496 million reads was generated from 12 samples. On average, 82% of reads were mapped to a unique location in the *M. capsulatus* Bath genome (Supplementary Figure S2 and Table S3). Principal component analysis demonstrated that cells grown on nitrate at 42°C (NMS42) and ammonia at 42°C and 37°C (AMS37, AMS42) were closely clustered and clearly separated from cells grown on nitrate medium at 37°C (NMS37). Changes in growth rate led to different transcript abundances in NMS37 as compared to NMS42, AMS37, and AMS42 (Figure 2). During growth on AMS medium at 42°C and 37°C, we did not observe a large change from growth on NMS medium at 42°C. This may reflect that the growth rate of *M. capsulatus* Bath on NMS42, AMS37, and AMS42 was similar (Figure 1 and Supplementary Table S2).

We next identified genes with altered abundance profiles during utilization of the different nitrogen sources at different temperatures. We chose the same time point (15 h) for RNA extraction for all the conditions. For analysis of the gene expression patterns during growth, nitrate at 37°C (NMS37) was chosen as the control.

The genome of *M. capsulatus* Bath contains 3,120 predicted coding sequences. We used a fold change >2 and an adjusted *p* value <0.05 as the cutoff for comparative gene expression. When *M. capsulatus* Bath was grown in AMS37, transcription of 140 genes was increased and 219 genes was decreased, while in AMS42, transcription of 725 genes was increased and 627 genes was decreased when compared with the growth in NMS37. In cells grown in NMS42, transcription of 571 genes was increased and that of 508 genes was decreased when compared to NMS37 (Supplementary Figure S3).

Gene ontology analysis was performed for the genes showing significantly higher or lower transcript abundance (value of *p* <0.05) in the cells grown in AMS37 vs. NMS37, NMS42 vs. NMS37, and AMS42 vs. NMS37. In cells grown in AMS37 vs. NMS37, 86 subcategories were identified based on gene ontology including respiration, transportation, assimilation and metabolism of nitrate and ammonia, methane monooxygenase complex, and amino acid biosynthesis (Supplementary Table S4). In cells grown in NMS42 vs. NMS37, gene transcription was subcategorized into 15 categories based on gene ontology terms. The significantly enriched terms based on gene count were related to respiration, tetrahydrofolate and folate biosynthesis, fatty acid and lipid biosynthesis, single carbon carrier biosynthesis, and amino acid biosynthesis (Supplementary Table S5). In cells grown in AMS42 vs. NMS37, gene transcription was subcategorized into 20 categories. The significantly enriched terms were related to respiration, nicotinamide adenine dinucleotide (NAD) biosynthesis and metabolism, fatty acid and lipid biosynthesis, cell structure biosynthesis, and histidine biosynthesis (Supplementary Table S6).

### 3.4. Transcriptional profile of the oxidative phosphorylation pathway

**Figure 3:**
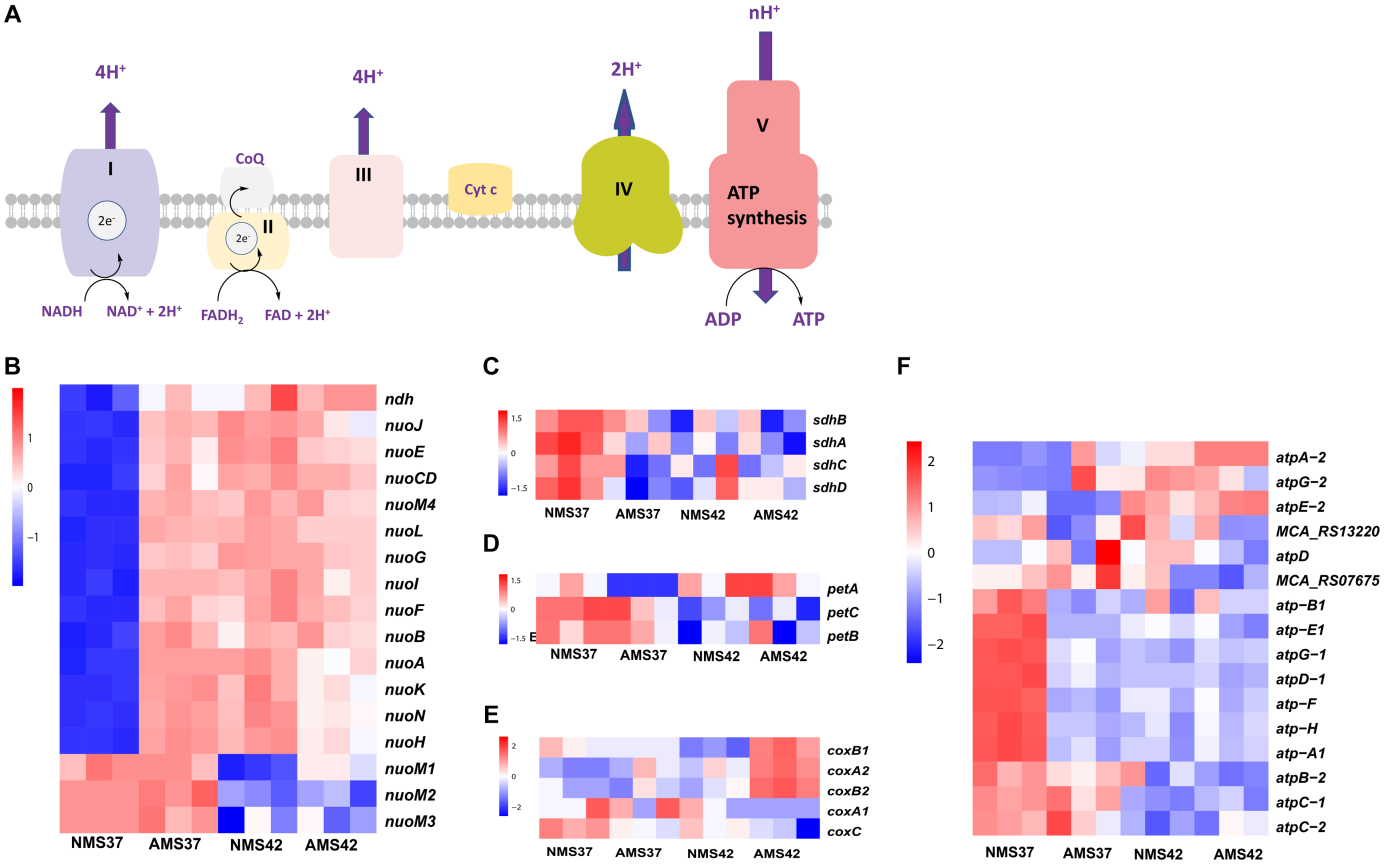
Transcriptional changes in oxidative phosphorylation pathway. Schematic representation of oxidative phosphorylation pathway **(A)** transcript abundance in the oxidative phosphorylation pathway complex I **(B)**, complex II **(C)**, complex III **(D)**, complex IV **(E)**, and complex V **(F)** in *M. capsulatus* Bath, grown on nitrate mineral salt medium at 42°C (NMS 42°C) and at 37°C (NMS 37°C), ammonium mineral salt medium at 42°C (AMS 42°C) and at 37°C (AMS 37°C). Samples were collected in an exponential phase at the 15 h time point. RNA-seq data was collected in triplicate for each condition. Color key represents the *z*-score for each gene (normalized for all growth conditions).

The electron transport chain of oxidative phosphorylation catalyzes the bacterial energy conversion process. We focused on the genes involved in the oxidative phosphorylation pathway (Figure 3). Regarding complex I, transcript abundance of 10 of the 14 genes was higher (>3 fold) in cells when grown on AMS37 vs. NMS37, NMS42 vs. NMS37, and AMS42 vs. NMS37 (Figure 3A and Supplementary Table S7). However, we did not observe any significant changes in the transcription of genes involved in complex II, III, and IV (Figures 3B–D and Supplementary Table S7).

From complex V, the transcript abundance of F0F1 complex subunits was higher in cells when grown on NMS37 vs. NM42, and AMS42 vs. NMS37. No significant fold changes in the transcript abundance of other genes were observed (Figure 3E and Supplementary Table S7).

### 3.5. Transcriptional profile of methane oxidation pathway

**Figure 4:**
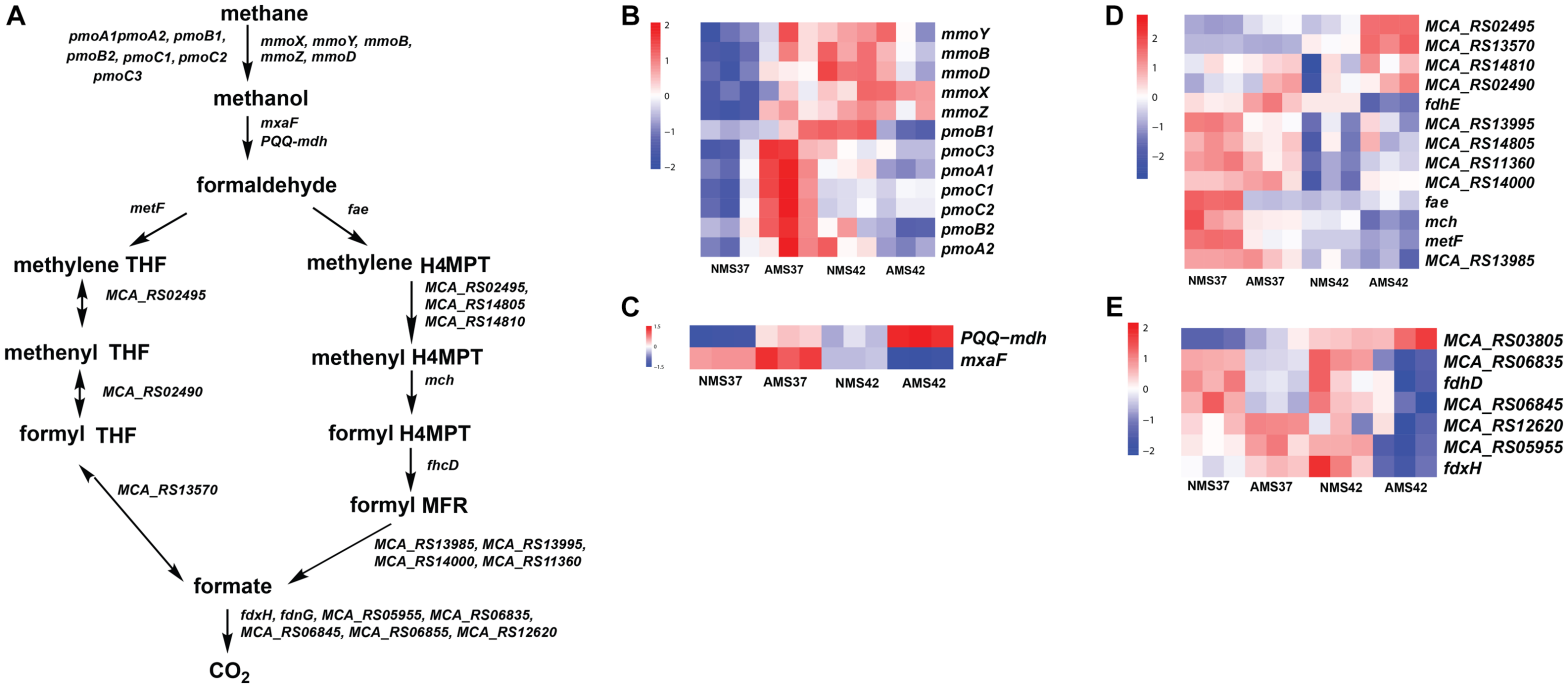
Methane oxidation pathway in *M. capsulatus* Bath **(A)**, transcript abundance in the methane oxidation pathway from methane to methanol **(B)**, methanol to formaldehyde **(C)**, formaldehyde to formate **(D)**, and formate to CO_2_ **(E)**, in *M. capsulatus* Bath grown on nitrate mineral salt medium at 42°C (NMS42) and at 37°C (NMS37), ammonium mineral salt medium at 42°C (AMS 42°C) and at 37°C (AMS 37°C). Color key represents the *z*-score for each gene (normalized for all growth conditions).

In the methane oxidation pathway, the transcript abundance of the four soluble methane monooxygenase (SMO) gene subunits was higher in cells when grown on AMS37 vs. NMS37, NMS42 vs. NMS37, and AMS37 vs. NMS37 (Figure 4B and Supplementary Table S8). An increase in transcript abundance of particulate methane monooxygenase (PMOI) subunits was also observed in AMS37 vs. NMS37.

From the methanol oxidation pathway, the higher transcript abundance of PQQ-dependent methanol dehydrogenase gene (PQQ-MDH) was observed on AMS37 vs. NMS37 and NMS42 vs. NMS37. When cells grown on AMS42 vs. NMS37 transcriptional abundance was increased by 10 fold, whereas the transcriptional abundance of other subunits of PQQ-dependent methanol dehydrogenase was decreased by 3 fold on AMS42 vs. NMS37. The transcript abundance of most of the genes involved in the methane oxidation metabolic pathway from formaldehyde to formate was higher in cells grown on NMS37, as compared to NMS42, and AMS42. The transcript abundance of all the genes involved in H4MPT pathway was higher in cells when grown on NMS42 vs. NMS37 and AMS42 vs. NMS37 (Figure 4C and Supplementary Table S8).

From the tetrahydrofolate (THF) pathway, the transcript abundance methylenetetrahydrofolate reductase was higher in cells when grown on NMS37 vs. AMS37, NMS37 vs. NMS42, and NMS37 vs. AMS42. We did not observe significant changes in the transcript abundance of other genes involved in the THF pathway (Figure 4D and Supplementary Table S8).

In the formate to CO_2_ pathway, transcript abundance of 3 of 7 subunits of formate dehydrogenase was higher in cells when grown on NMS37 vs. AMS42 (Figure 4E and Supplementary Table S8). We did not observe any significant change in the transcript abundances of genes in other tested conditions.

### 3.6. Transcriptional profile of nitrogen cycle

**Figure 5:**
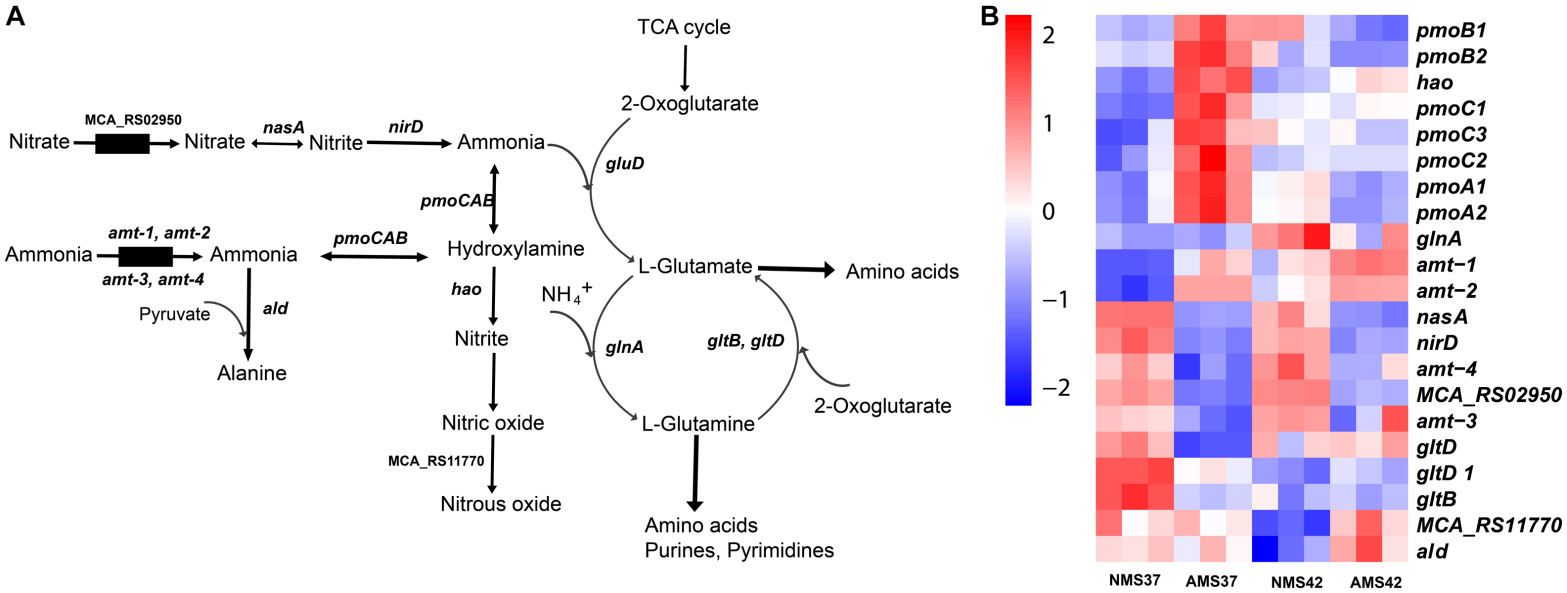
Transcriptional changes in nitrogen metabolism in *M. capsulatus* Bath. Nitrogen pathway in *M. capsulatus* Bath **(A)**, transcript abundance in nitrogen assimilation and metabolism genes in *M. capsulatus* Bath **(B)**, grown on nitrate mineral salt medium at 42°C (NMS 42°C) and at 37°C (NMS 37°C), ammonium mineral salt medium at 42°C (AMS 42°C) and at 37°C (AMS 37°C). Color key represents the *z*-score for each gene (normalized for all growth conditions).

We next evaluated how *M. capsulatus* Bath responds to nitrate and ammonium as nitrogen sources in the medium. During nitrate consumption, the transcript abundance of the nitrate transporter was significantly lower in cells when grown on AMS37 vs. NMS37 (74 fold), and AMS42 vs. NMS37 (17 fold) (Figure 5 and Supplementary Table S9). Both the subunits of nitrate reductase (*nasA*, *nirD*) showed lower transcript abundance in cells when grown on NMS42 vs. NMS37, while in AMS37 vs. NMS37 the decrease in transcript abundance of both the subunits was 12 fold, and in AMS42 vs. NMS37 at least a 10 fold decrease was observed in the transcript abundance of both subunits. We did not observe any significant change in the transcript abundance of genes responsible for the conversion of nitric oxide to nitrous oxide. Both subunits of glutamate synthase showed a decrease in transcript abundance in cells when grown on NMS42 vs. NMS37. In cells grown on AMS42 vs. NMS37, decrease in transcript abundance of beta subunit of glutamate synthase was observed.

The transcript abundance of 2 of the 4 ammonia transporters (*amt-1*, *amt-2*) was higher in cells when grown on AMS37 vs. NMS37, NMS42 vs. NMS37, and AMS42 vs. NMS37. The transcript abundance of the other 2 transporters was higher in cells when grown on NMS37 vs. AMS37. From the genes responsible for the oxidation of ammonia to hydroxylamine, transcript abundance of 3 of the 7 genes was higher in AMS37 vs. NMS37, and transcript abundance of 2 of the 7 genes was higher in AMS42 vs. NMS37. The transcript abundance of hydroxylamine reductase was higher in cells when grown on AMS37 vs. NMS37, and AMS42 vs. NMS37. The transcript abundance of alanine dehydrogenase was higher in cells when grown on AMS37 vs. NMS37 and AMS42 vs. NMS37 (Figure 5). The transcript abundance of GS/GOGAT pathway genes was lower in cells when grown on AMS37 vs. NMS37, and AMS42 vs. NMS37.

### 3.7. Quantitative PCR validation of differential gene expression

**Figure 6:**
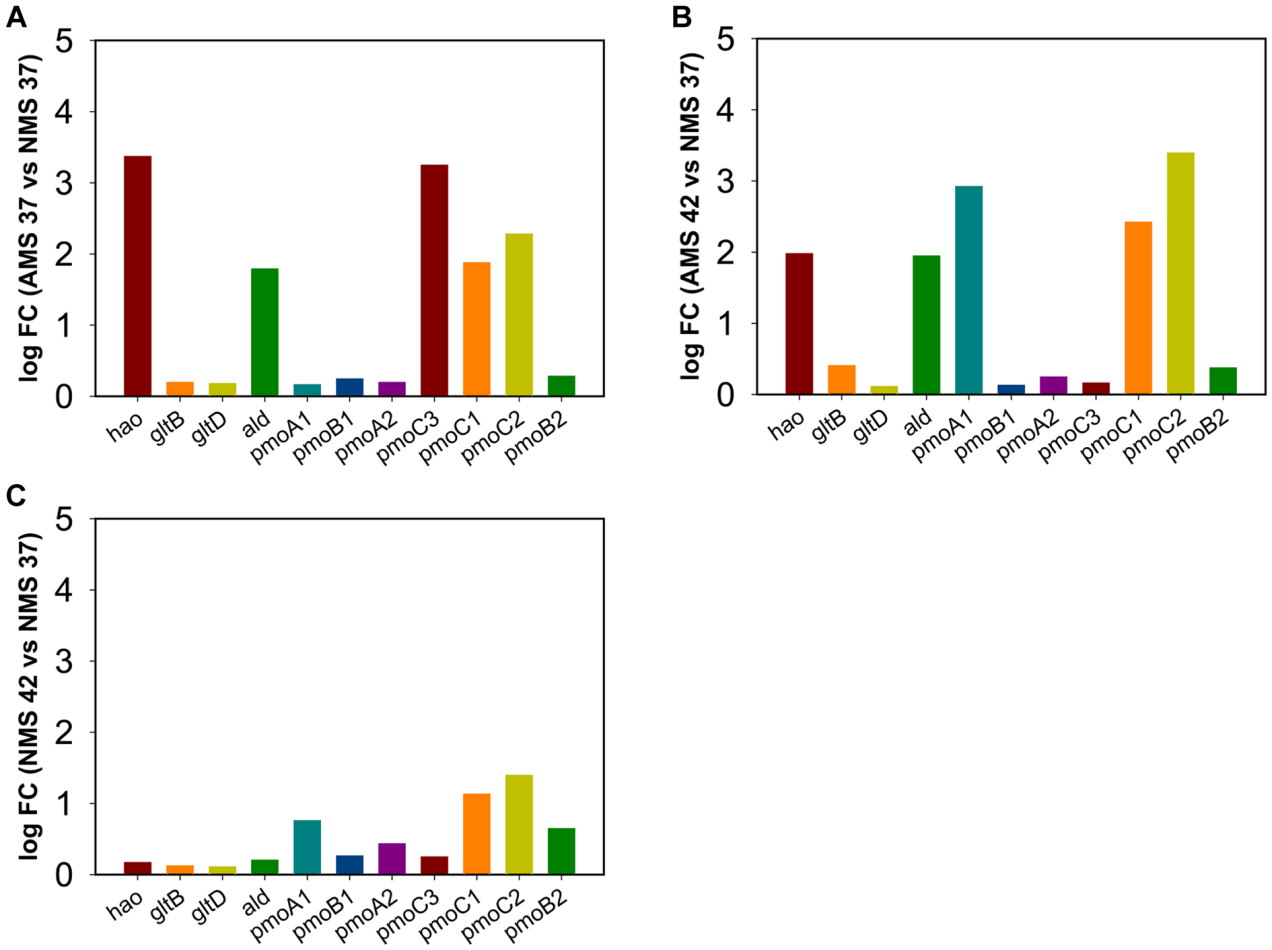
Quantitative PCR validation of differentially expressed genes in *M. capsulatus* Bath. The relative expression fold change for genes in AMS37 vs. NMS37 **(A)**, AMS42 vs. NMS37 **(B)**, and NMS42 vs. NMS37 **(C)**. *M. capsulatus* Bath grown on nitrate mineral salt medium at 42°C (NMS 42°C) and at 37°C (NMS 37°C), ammonium mineral salt medium at 42°C (AMS 42°C) and at 37°C (AMS 37°C). Data was collected in biological triplicates for each condition. Gene expression levels were normalized based on the expression of the DNA directed RNA polymerase (*rpoA*) gene. Hydroxylamine reductase (*hao*), hydroxylamine oxidation protein (*haoB*), glutamate synthase large subunit (*gltB*), glutamate synthase small subunit (*gltD*), alanine dehydrogenase (*ald*), particulate methane monoxygenases/ammonia monoxygenase subunit C3 (*pmoC3*), particulate methane monoxygenases/ammonia monoxygenase subunit B1 (*pmoB1*), subunit A1 (*pmoA1*), subunit C1 (*pmoC1*), subunit B2 (*pmoB2*), subunit A2 (*pmoA2*), and subunit C2 (*pmoC2*).

To confirm results obtained using RNA-seq analysis, we examined the expression of genes involved in the nitrogen metabolism pathway using quantitative PCR during growth on AMS and NMS medium at 42 and 37°C (Figure 6). The *rpoA* gene encoding the alpha subunit of RNA polymerase (MCA_11500) was used as a housekeeping gene to compare relative expression levels (Supplementary Table S1 and Figure S4). From the *pmoCAB* gene cluster encoding ammonia monooxygenases/particulate methane monooxygenases, expression of 3 of the 7 genes was higher in cells grown on AMS37 vs. NMS37 (*pmoC3, pmoC1, pmoC2*) (Figure 6A) and AMS42 vs. NMS37 (*pmoA1, pmoC1, pmoC2*) (Figure 6B). We did not observe a significant change in the expression of *pmoCAB* genes in cells grown on NMS42 vs. NMS37 (Figure 6C). We found that expression of hydroxylamine reductase, the gene responsible for hydroxylamine to nitrite conversion, was higher in cells grown on AMS37 vs. NMS37 and AMS42 vs. NMS37, while in cells grown on NMS42 vs. NMS37 no significant change in expression of this gene was observed. We did not observe any change in the expression of the glutamate synthase genes in any of the conditions tested. The expression of alanine dehydrogenase was increased in cells grown on AMS37 vs. NMS37 and AMS42 vs. NMS37, while in cells grown on NMS42 vs. NMS37 no significant change in alanine dehydrogenase was observed (Figure 6). Altogether, the qPCR data supports the RNA-seq data in that nitrate and ammonium medium differentially affected gene expression in *M. capsulatus* Bath.

### 3.8. Metabolomic analysis of *M. capsulatus* Bath

**Figure 7:**
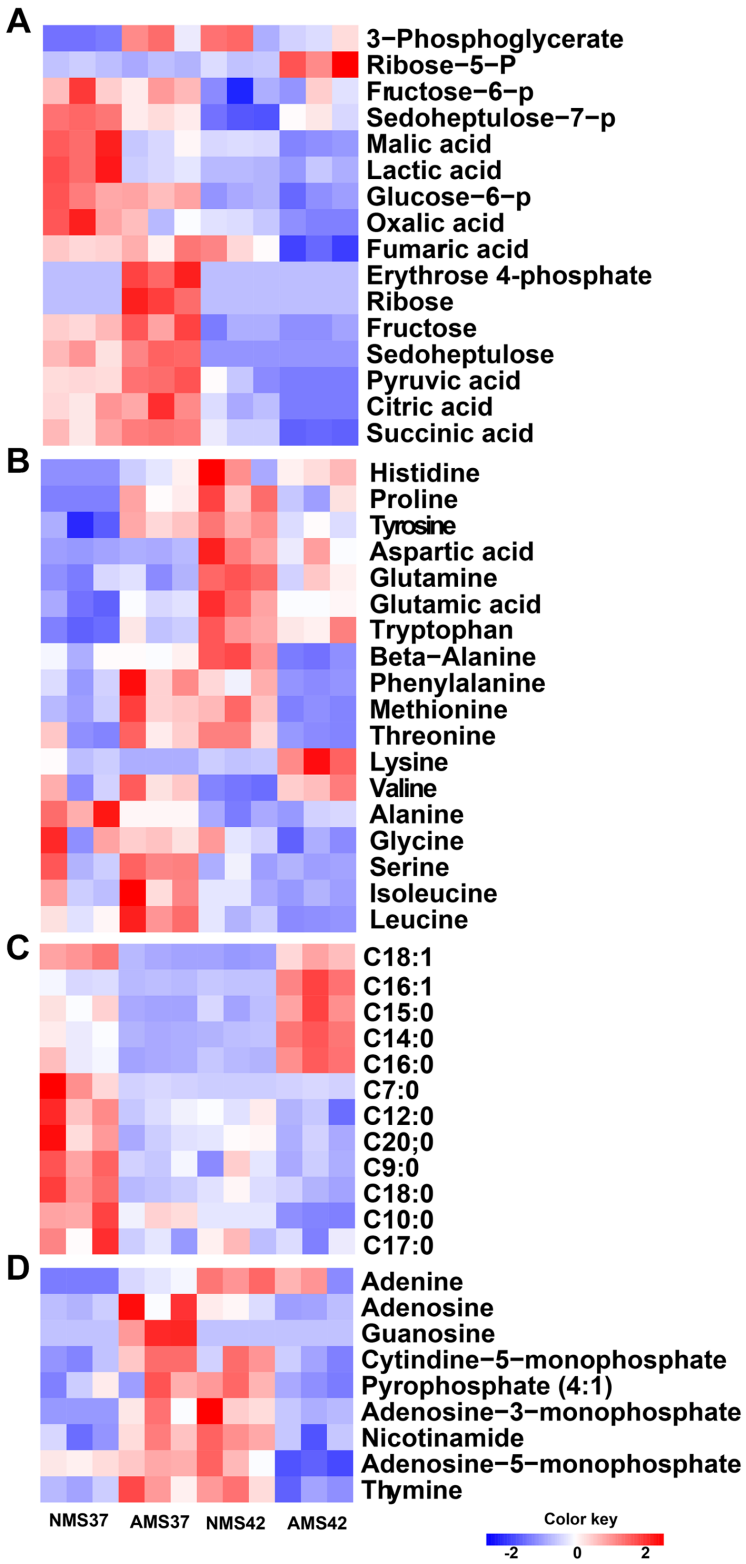
Heat map analysis of the intracellular metabolome. Heat map of intracellular metabolites C1 assimilation pathway **(A)**, amino acids **(B)**, fatty acid composition **(C)**, and nitrogen bases **(D)** of *M. capsulatus* Bath grown on nitrate mineral salt medium at 42°C (NMS 42°C) and at 37°C (NMS 37°C), ammonium mineral salt medium at 42°C (AMS 42°C), and at 37°C (AMS 37°C). Data was collected in biological triplicates for each condition. Color key represents the *z*-score for each gene (normalized for all growth conditions).

We next compared the relative concentrations of 57 intracellular metabolites, including amino acids, intermediates of the central carbon metabolism pathway, nitrogen bases, and fatty acid composition, produced by cells grown on NMS37 vs. NMS42, AMS37, and AMS42 (Figure 7).

When comparing amino acids, we observed an increase in relative concentration of amino acids produced during the pentose phosphate pathway, glycolysis and TCA cycle in cells grown on AMS37 vs. NMS37, NMS42 vs. NMS37, and AMS42 vs. NMS37. From the central carbon metabolism pathway, an increase in relative concentration of 6 of the 16 intermediates was observed in cells grown on AMS37 vs. NMS37. The relative concentrations of 3-phosphoglycerate and ribose-5-phosphate were increased in cells grown on NMS42 vs. NMS37 and AMS42 vs. NMS37. From fatty acid composition, the increase in relative concentration of 4 long chain fatty acids was observed only in cells grown on AMS42 vs. NMS37. The relative concentration of nitrogen bases was increased in NMS42 vs. NMS37 and AMS37 vs. NMS37 (Figure 7).

## 4. Discussion

Multi-omics approaches can be used to determine the active metabolic pathways and how cells adapt and respond to changing environments (Nguyen et al., 2019). In this study, we systematically investigated the effect of nitrate and ammonium as nitrogen sources as well as the impact of temperature on growth dynamics of industrially important methanotroph *M. capsulatus* Bath through growth studies, carbon and nitrogen source consumption, transcriptomics and metabolomic analyses. We tested whether growth would be enhanced by replacing nitrate with ammonia at 37°C or 42°C. These temperatures were chosen because several studies have reported 42°C as the optimum temperature for the growth of *M. capsulatus* Bath (Troup et al., 1994; Kalyuzhnaya and Xing, 2018). However, some studies have found that *M. capsulatus* Bath can grow optimally at 37°C (Foster and Davis, 1966; Soni et al., 1998; Henard et al., 2018). Another factor that likely influences growth rate is that gas solubility increases with a decrease in temperature. Therefore, we tested the effect of temperature downshift on the growth profile of *M. capsulatus* Bath as compared to 42°C. We discovered temperature dependent responses when we focused on effects specific to nitrogen source used. *M. capsulatus* Bath showed lack of preference when grown on either nitrogen source at 42°C. However, a notable difference in growth rate and doubling time was observed at 37°C. At 37°C, *M. capsulatus* Bath grew slightly faster than ammonium over nitrate, resulted in a higher growth rate and reduced doubling time. This preference for ammonium at lower temperature suggests the adaptive strategy of *M. capsulatus* Bath for optimal growth. This is consistent with previous findings in the γ-proteobacterium *Methylomonas denitrificans*, sp. nov. type strain FJG1 when grown on NMS and AMS medium (Kits et al., 2015). A key observation from our gas consumption studies was that, regardless of the nitrogen source and temperature provided, all the cultures entered stationary phase after 24 h despite the presence of methane in the gas phase, indicating a common metabolic switch in response to the limitation of oxygen. *M. capsulatus* Bath cells may have entered a stationary phase due to oxygen depletion. This finding suggests that oxygen availability plays an important role in regulating the growth dynamics of *M. capsulatus* Bath (Dunfield et al., 2007; Islam et al., 2008; Guerrero-Cruz et al., 2021).

The observed growth differences were further validated by analyzing nitrate and ammonia utilization pattern of *M. capsulatus* Bath. When grown in AMS medium, *M. capsulatus* Bath showed comparable ammonia utilization at both 37°C and 42°C, showing a consistent ammonia utilization capacity irrespective of temperature fluctuations. The consistency in ammonia utilization supports the growth rate in AMS medium, irrespective of temperature shifts. On the other hand, enhanced nitrate utilization rates were observed in NMS medium at 42°C, indicating a higher affinity for nitrate at the higher temperature. These findings highlight the complex interplay between nitrogen source availability, temperature, and growth dynamics. The flexibility of *M. capsulatus* Bath to alter the nitrogen utilization strategies in response to nitrogen source and temperature highlights its adaptability to diverse environmental conditions.

Also, the concentration of nitrite, a cytotoxic product of nitrate reduction was higher in the medium with nitrate as compared to cells grown on ammonia at both 42°C and 37°C, supporting the physiological advantage of using ammonia for the growth of *M. capsulatus* Bath. Ammonia addition allows higher nitrogen assimilation and might reduce metabolic energy requirements as compared to nitrate. This would allow the allocation of the remaining energy to other cellular processes, such as growth (Lachmann et al., 2019).

The most common significantly enriched gene ontology term in our experiments was related to respiration. Therefore, we measured the abundance of genes involved in oxidative phosphorylation. The enzyme complexes I, III, and IV of the bacterial respiratory chain catalyze the bacterial energy conversion process. Complex I catalyzes NADH oxidation by quinone, complex III catalyzes quinol oxidation by cytochrome c and complex IV catalyzes cytochrome c oxidation by O_2_. Complex I is an entry point in the electron transport chain (Lesner et al., 2022). In *E. coli*, of the 14 subunits of complex I, subunits *NuoL*, *NuoM*, and *NuoN* subunits are responsible for pumping one proton each, whereas the fourth proton is believed to be pumped via the *NuoH*, *NuoA*, *NuoJ*, and *NuoK* subunits (Kaila and Wikström, 2021). For the oxidative phosphorylation pathway, complex I exhibited substantial upregulation in 10 of 14 genes, with at least a 3-fold increase in transcript abundance during growth of *M. capsulatus* Bath cells on ammonium at both 37°C and 42°C compared to nitrate at 37°C. This increased activity of complex I, indicates metabolic adaptation of *M. capsulatus* Bath cells to utilize ammonia as a nitrogen source. Interestingly, no significant changes were observed in the transcription of genes involved in complex II, III, and IV, suggesting a specific response pattern within the oxidative phosphorylation pathway. Notably, the increase in complex I gene expression coincided with faster cell growth, supporting a close connection between energy production and growth rate. The remarkable increase in the transcript levels of complex I genes suggests the additional requirement of ATP synthesis by faster growing cells in nitrate at 42°C and ammonia at 42 and 37°C (Hua et al., 2004; Kühn et al., 2015).

*M. capsulatus* Bath possesses methane monooxygenase in both particulate membrane-bound form and a soluble form. A higher transcript abundance of the *pmoCAB* cluster was observed in AMS37 vs. NMS37. This suggests that the transcription of *pmoCAB* genes was induced by the addition of ammonia at 37°C. Also, the *mmo* gene cluster showed higher transcript abundance in cells grown on NMS42 vs. NMS37 and AMS42 vs. NMS37, suggesting induced transcription of *mmo* genes at 42°C.

*M. capsulatus* Bath can oxidize the methanol produced from methane using methanol dehydrogenase. A significant increase in the transcription abundance of *PQQ-mdh* in AMS37, AMS42, and NMS37 conditions suggests that the methanol oxidation rate was higher in these conditions as compared to NMS37. The differential regulation of methane monooxygenase genes in response to ammonia and temperature highlights the flexibility of methane oxidation pathways, potentially affording *M. capsulatus* Bath a competitive advantage in diverse niches.

In methanotrophs, the preferred nitrogen source is strain specific. Nitrogen can be utilized by several mechanisms including nitrogen fixation, reduction of nitrate to ammonia (Murrell and Dalton, 1983), and oxidation of ammonia to nitrite (Dalton, 1977; King and Schnell, 1994; Nyerges and Stein, 2009). *M. capsulatus* Bath possesses the alanine dehydrogenase (*ald*), and glutamate dehydrogenase (*gluD*), glutamine synthatase/glutamate synthase (*glnA*/*gltB*, *gltD*) (*GS-GOGAT*) pathways for ammonia assimilation. Under low ammonium in environmental or medium conditions (<0.5 mM) or in a medium containing nitrate as a nitrogen source, *M. capsulatus* Bath uses the GS-GOGAT pathway for ammonia assimilation into glutamate. In contrast, under high ammonia conditions, it assimilates ammonia using alanine dehydrogenase (Murrell and Dalton, 1983). We examined some key genes involved in the nitrogen cycle of *M. capsulatus* Bath. During nitrate consumption, there was significant downregulation of nitrate transporters and nitrate reductase subunits in cells grown in AMS medium, especially at 37°C, indicates a substantial decrease in nitrate assimilation. This downregulation might be due to reduced need for nitrate uptake and assimilation in presence of ammonium. Interestingly, no significant changes in genes responsible for nitric oxide to nitrous oxide conversion irrespective of the nitrogen source suggests a potential pathway for this conversion. In contrast, in cells grown in AMS medium, the upregulation of certain transporters and genes involved in oxidation of ammonia to hydroxylamine suggests an enhanced capacity to consume ammonium. This observation aligns with the preference for ammonium as a nitrogen source under changing temperature. Further, the increased transcript abundance of hydroxylamine reductase and alanine dehydrogenase underscores the bacterium’s ability to convert ammonia to nitrite and alanine, reflecting an active nitrogen assimilation pathway. The validation of RNA-seq data through quantitative PCR further strengthens the reliability of these findings. The higher expression of genes encoding ammonia monooxygenases/particulate methane monooxygenases and hydroxylamine reductase in cells grown on ammonium supports the RNA-seq data, confirming the enhanced capacity of *M. capsulatus* Bath for ammonium oxidation and assimilation in ammonium-rich medium. The lack of significant changes in the expression of glutamate synthase genes under different conditions suggests a stable glutamate synthesis pathway, possibly highlighting a consistent requirement for this essential amino acid in different environments.

In cells grown on ammonium at 42°C (AMS42 vs. NMS37), the observed increase in relative concentrations of long-chain fatty acids relates with the upregulation of genes in the oxidative phosphorylation pathway. This increase suggests a link between enhanced fatty acid metabolism and increased energy production through oxidative phosphorylation, indicating a coordinated response to boost energy production. Interestingly, differential expression of genes involved in nitrogen assimilation pathways correlate with the changes in the relative concentrations of nitrogen bases. The metabolomic data demonstrates increased nitrogen base concentrations in cells grown on nitrate at 42°C (NMS42 vs. NMS37) and ammonium at 37°C (AMS37 vs. NMS37). This correlates with the downregulation of nitrate assimilation genes (e.g., nitrate transporter and nitrate reductase subunits) in cells grown in AMS medium (AMS37 vs. NMS37 and AMS42 vs. NMS37). These coordinated changes in gene expression and metabolite concentrations emphasize the ability of *M. capsulatus* Bath to adjust metabolic pathways to efficiently use different nitrogen sources and cope with temperature variations.

## 5. Conclusion

In conclusion, this study provides valuable insights into the metabolic flexibility and adaptive strategies of the model methanotroph *M. capsulatus* Bath. We systematically studied the effect of nitrate and ammonium as nitrogen sources and temperature variations on the growth dynamics, gene expression patterns, and metabolite profile of *M. capsulatus* Bath. Our results revealed temperature-dependent responses of *M. capsulatus* Bath as ammonium was found to be a preferred nitrogen source over nitrate at 37°C. This preference emphasizes the adaptive strategy of *M. capsulatus* Bath for optimal growth under specific temperature conditions. Moreover, our results showcase the complex interplay between temperature, nitrogen source availability, and growth dynamics showing its ability to modify nitrogen utilization strategies in response to changing environmental conditions. The upregulation of key genes involved in the oxidative phosphorylation pathway, central carbon metabolism pathways, nitrogen metabolism coupled with metabolite concentrations, showcases the ability of *M. capsulatus* Bath to regulate its metabolism for energy production and synthesis of precursor metabolites. Overall, this study provide a detailed understanding of *M. capsulatus* Bath’s metabolic responses to varying environmental conditions, offering valuable insights for both microbial ecology research and biotechnological applications.

## Data availability statement

The datasets presented in this study can be found in online repositories. The names of the repository/repositories and accession number(s) can be found in the article/Supplementary Material.

## Author contributions

AB: Conceptualization, Data curation, Investigation, Methodology, Software, Validation, Visualization, Writing – original draft, Writing – review & editing. AD: Methodology, Software, Visualization, Formal analysis, Writing – review & editing. SJ: Formal analysis, Methodology, Validation, Writing – review & editing. DP: Project administration, Resources, Supervision, Writing – review & editing. PL: Project administration, Resources, Supervision, Validation, Writing – review & editing. RM: Conceptualization, Formal analysis, Funding acquisition, Resources, Supervision, Validation, Writing – review & editing. CR: Conceptualization, Formal analysis, Funding acquisition, Investigation, Project administration, Resources, Supervision, Validation, Visualization, Writing – original draft.
