# Supplementary material for: Transcriptional and metabolomic responses of Methylococcus capsulatus Bath to nitrogen source and temperature downshift
Source: Front Microbiol. 2023 Oct 20;14:1259015. doi: 10.3389/fmicb.2023.1259015 (PMC10623323; doi:10.3389/fmicb.2023.1259015)
Supplement: Supplementary file 1 [file Data_Sheet_1.docx]

***Supplementary Material***

**Transcriptional and metabolomic responses of *Methylococcus capsulatus* Bath to nitrogen source and temperature downshift**

Ashwini Ashok Bedekar^1,2,3^, Anshu Deewan^2,3^, Sujit Sadashiv Jagtap^2,3^, David A. Parker^1,5^, Ping Liu^1,5^, Roderick I. Mackie^1,2,4^ and Christopher V. Rao^1,2,3*^

^1^ Energy & Biosciences Institute, Materials Research Laboratory, University of Illinois at Urbana-Champaign, IL, 61801, USA

^2^ Carl R. Woese Institute for Genomic Biology, University of Illinois at Urbana-Champaign, IL 61801, USA

^3^ Department of Chemical and Biomolecular Engineering, University of Illinois at Urbana-Champaign, IL 61801, USA

^4^ Department of Animal Sciences, University of Illinois at Urbana-Champaign, IL 61801, USA

^5^ Shell Exploration and Production Inc., Westhollow Technology Center, Houston, TX 77082-3101, USA

*Corresponding author:

E-mail: [cvrao@illinois.edu](mailto:cvrao@illinois.edu).

Phone: (217) 244-2247. Fax: (217) 333-5052.

Address: 600 S. Mathews Ave., Urbana, IL 61801, USA.


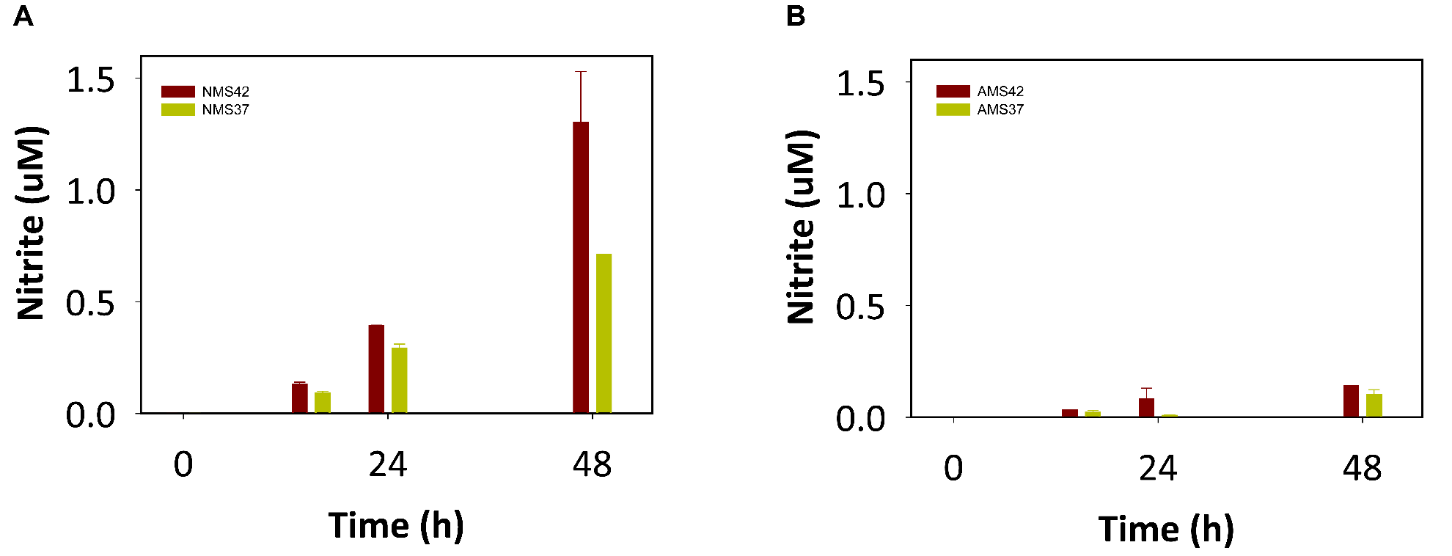


**Supplementary Figure S1.** Nitrite production in *M. capsulatus* Bath cells grown on (A) nitrate mineral salt medium at 42 ^o^C (NMS42) and 37 ^o^C (NMS37) and (B) ammonium mineral salt medium at 42 ^o^C (AMS42) and 37 ^o^C (AMS37).


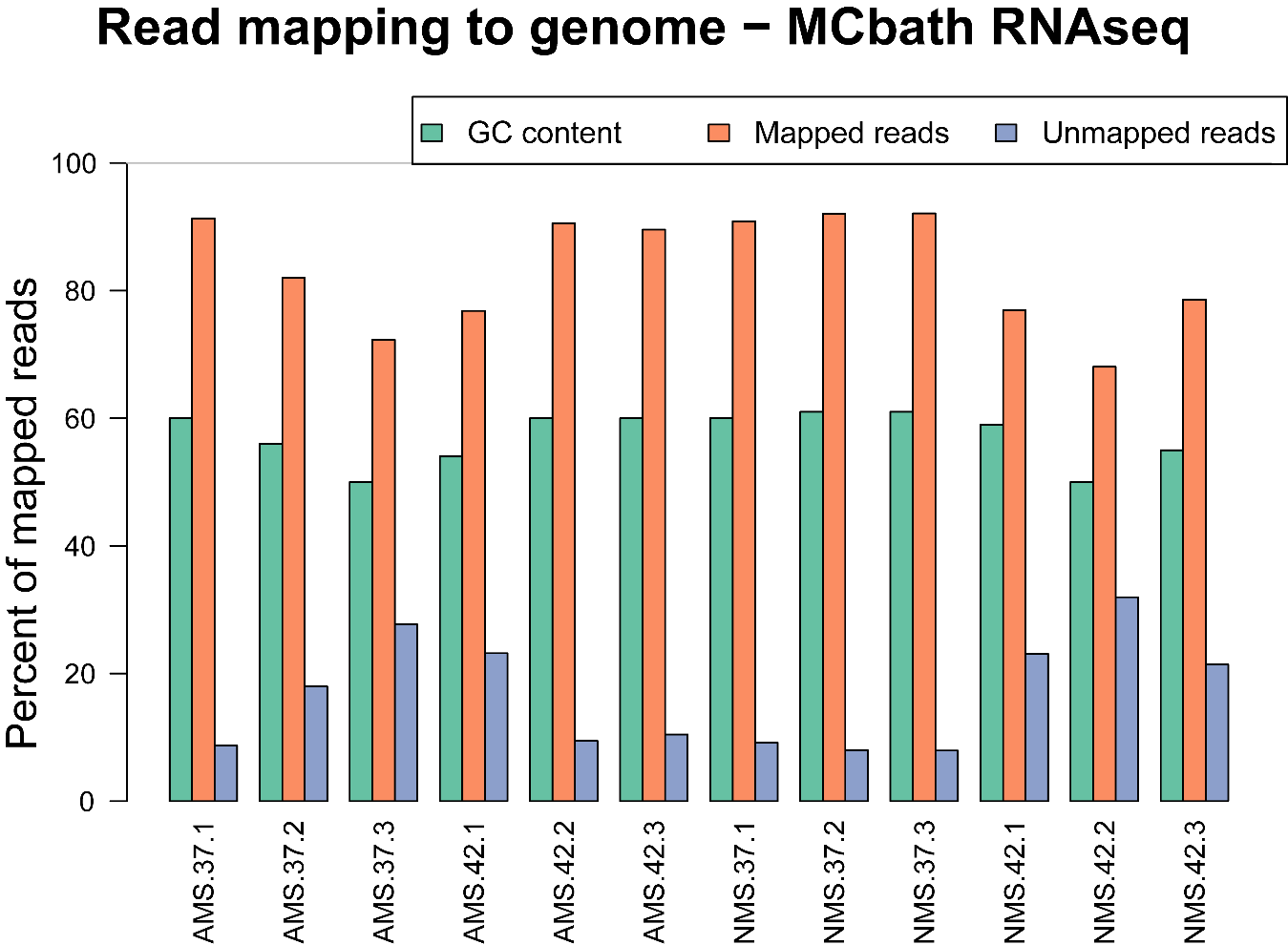


**Supplementary Figure S2.** Summary of mapped reads from the RNA-seq data analysis of *M*. *capsulatus* Bath. The x-axis is transcriptomes from different conditions and replicates and y-axis is the percentage of reads in each sample. The raw reads were filtered for adapter sequencing and low-quality reads (QCfiltered) and mapped to the *M*. *capsulatus* Bath reference genome. Most reads mapped to a unique gene location (in a gene), and a small fraction was mapped to non-coding regions (not in gene) and to multiple locations in the genome (multimapped).


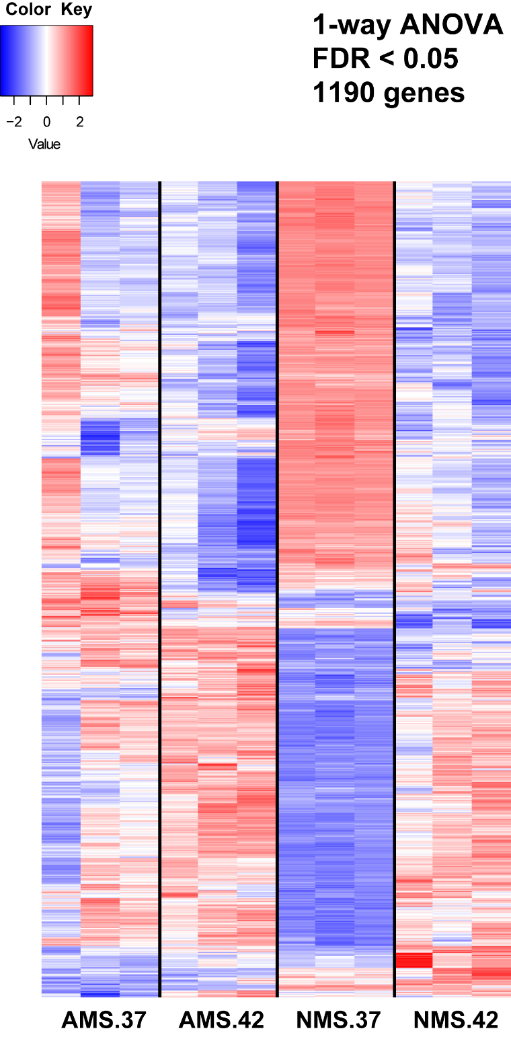


**Supplementary Figure S3.** Heatmap generated from gene expression profiles of *M*. *capsulatus* Bath grown on ammonium at 37 ^o^C (AMS.37) and 42 ^o^C (AMS.42), and nitrate at 37 ^o^C (NMS.37) and 42 ^o^C (NMS.42). RNA-seq data was collected in triplicate for each condition. Color key represents the z-score for each gene (normalized for all growth conditions).


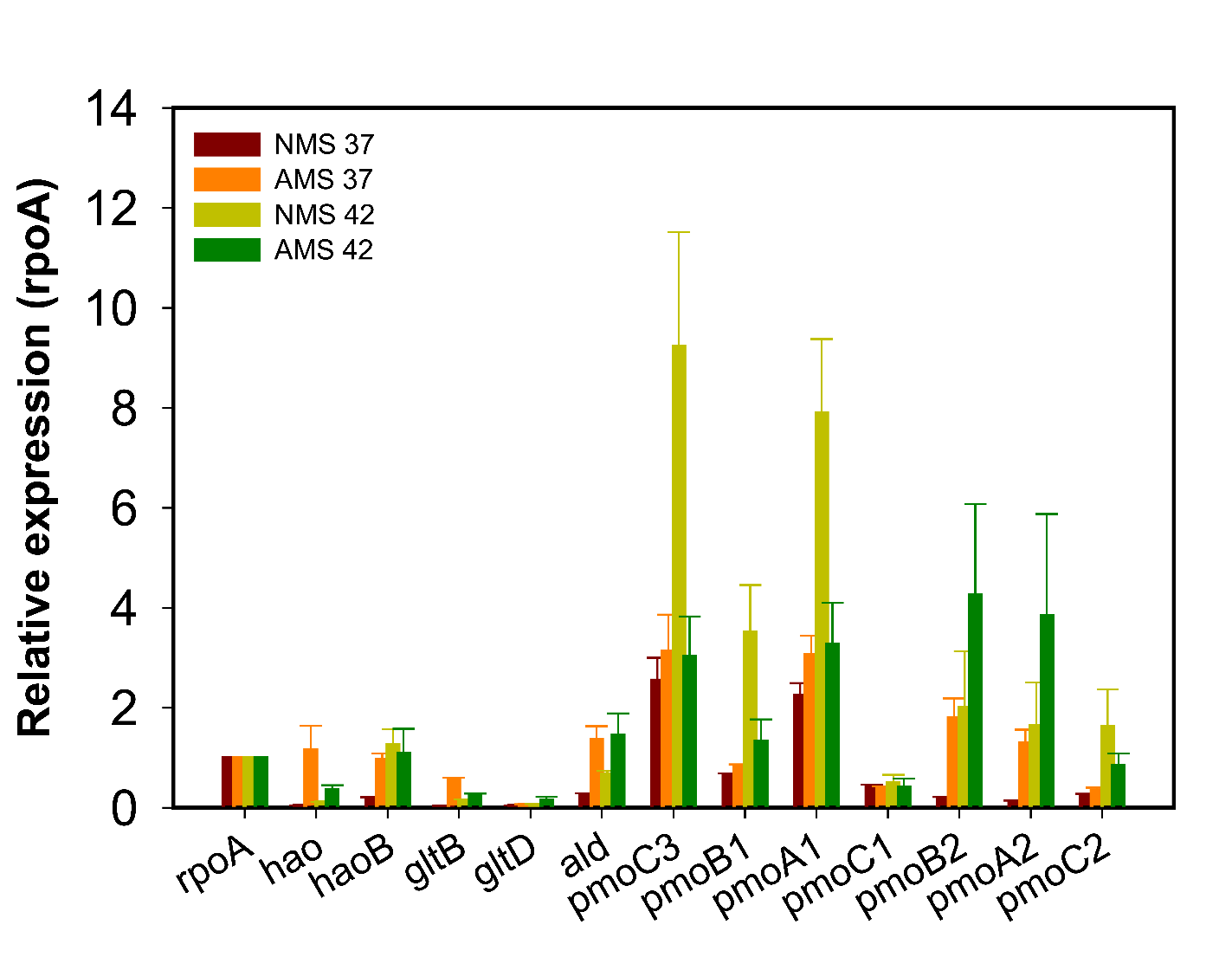


**Supplementary Figure S4.** Expression profiles for nitrogen source assimilation genes in *M*. *capsulatus* Bath. Gene expression levels were normalized based on the expression of the DNA directed RNA polymerase (*rpoA*) gene. *hao* hydroxylamine reductase, *haoB* hydroxylamine oxidation protein, *gltB* glutamine synthase large subunit, *gltD* glutamine synthase small subunit, *ald* alanine dehydrogenase, *pmoC3* particulate methane monoxygenases/ammonia monoxygenase subunit C3, *pmoB1* particulate methane monoxygenases/ammonia monoxygenase subunit B1, *pmoA1* particulate methane monoxygenases/ammonia monoxygenase subunit A1, *pmoC1* particulate methane monoxygenases/ammonia monoxygenase subunit C1, *pmoB2* particulate methane monoxygenases/ammonia monoxygenase subunit B2, *pmoA2* particulate methane monoxygenases/ammonia monoxygenase subunit A2, and *pmoC2* particulate methane monoxygenases/ammonia monoxygenase subunit C2.

**Supplementary Table S1** Oligonucleotides used in this study.

| **Sequence Name** | **Gene name** | **Sequence** |
| --- | --- | --- |
| MCA_RS04695 FWD | hydroxylamine reductase | GCT CAA GGA CAA GTA CGA GAA G |
| MCA_RS04695 REV |  | CGT GGG CGG TTG ATA GAA AT |
| MCA_RS01450 FWD | methane monooxygenase/ammonia monooxygenase subunit C | GGT GCG AGA AGC TGT AGA AG |
| MCA_RS01450 REV |  | CAC CTT CTG GTT CAT GGA AGA |
| MCA_RS04690 FWD | hydroxylamine oxidation protein HaoB | CGT GGG CGG TTG ATA GAA AT |
| MCA_RS04690 REV |  | GCT CAA GGA CAA GTA CGA GAA G |
| MCA_RS09975 FWD | glutamate synthase large subunit | CGC CAC ATC ACG ACA TCT AT |
| MCA_RS09975 REV |  | ACC CAC TTC CGA TAC CAA TTT |
| MCA_RS02795 FWD | alanine dehydrogenase | CTC GTG GTC AAG GTG AAA GA |
| MCA_RS02795 REV |  | GCC AGA TGG AAG AAG GTG AAT A |
| MCA_RS08835 FWD | methane monooxygenase/ammonia monooxygenase subunit A | AGT GCC ACA TGA AGT AGA TCA G |
| MCA_RS08835 REV |  | GTA CAT CCG CAT GGT AGA GAA G |
| MCA_RS08830 FWD | methane monooxygenase/ammonia monooxygenase subunit B | AGA AGG CGT GCC AGA AAT AG |
| MCA_RS08830 REV |  | GGA AGG CTC CAT GAG TGA AT |
| MCA_RS13975 FWD | methane monooxygenase/ammonia monooxygenase subunit A | AGT GCC ACA TGA AGT AGA TCA G |
| MCA_RS13975 REV |  | GTA CAT CCG CAT GGT AGA GAA G |
| MCA_RS09980 FWD | glutamate synthase subunit beta | TTG GCC GGC ATC CAT TT |
| MCA_RS09980 REV |  | CCT TCT GCT TCG TCT ATC GTA TC |
| MCA_RS13980 FWD | methane monooxygenase/ammonia monooxygenase subunit C | CCT GAG CGA AGC TGT AGA AG |
| MCA_RS13980 REV |  | CAC CTT CTG GTT CAT GGA AGA |
| MCA_RS08840 FWD | methane monooxygenase/ammonia monooxygenase subunit C | CCT GAG CGA AGC TGT AGA AG |
| MCA_RS08840 REV |  | CAC CTT CTG GTT CAT GGA AGA |
| MCA_RS13970 FWD | methane monooxygenase/ammonia monooxygenase subunit B | AGA AGG CGT GCC AGA AAT AG |
| MCA_RS13970 REV |  | GGA AGG CTC CAT GAG TGA AT |
| MCA_RS11500 FWD | DNA-directed RNA polymerase subunit alpha | GTC GAC CAG GAT AGC AAG ATG |
| MCA_RS11500 REV |  | ACC GGT TGG TTA TCG AGT TG |

**Supplementary Table S2.** Growth rates of *M. capsulatus* Bath in different combinations of nitrogen sources and temperature reported as changes in optical density (600 nm) per hour

|  | NMS 42 ^o^C | NMS 37 ^o^C | AMS 42 ^o^C | AMS 37 ^o^C |
| --- | --- | --- | --- | --- |
| Growth rate (h^-1^) | 0.20 ± 0.03 | 0.17± 0.01 | 0.20± 0.09 | 0.19± 0.01 |
| Doubling time | 3.47 | 4.16 | 3.40 | 3.71 |

**Supplementary Table S3.** Composition of total raw reads and summary of RNA-seq samples mapped to *M*. *capsulatus* Bath genome

| Sample | Total reads | Trimmed reads | Number of reads mapped to: | | | |
| --- | --- | --- | --- | --- | --- | --- |
|  |  |  | Unmapped | Not in gene | Ambiguous | Single gene |
| NMS37.1 | 44,520,859 | 298 | 4,073,134 | 5,185,596 | 1,561,709 | 33,771,814 |
| NMS37.2 | 40,111,712 | 153 | 3,190,254 | 4,667,389 | 1,447,182 | 30,867,668 |
| NMS37.3 | 37,662,760 | 171 | 2,980,108 | 4,279,816 | 1,311,072 | 29,154,311 |
| AMS37.1 | 52,867,310 | 286 | 4,607,881 | 5,072,306 | 1,744,505 | 41,544,640 |
| AMS37.2 | 41,697,202 | 278 | 7,493,115 | 4,587,656 | 953,594 | 28,789,607 |
| AMS37.3 | 42,642,147 | 305 | 11,804,596 | 4,349,282 | 859,593 | 25,790,831 |
| NMS42.1 | 41,317,872 | 266 | 9,536,764 | 5,125,187 | 883,321 | 25,859,055 |
| NMS42.2 | 37,760,898 | 299 | 12,065,742 | 3,875,493 | 677,049 | 21,282,762 |
| NMS42.3 | 38,600,759 | 256 | 8,260,774 | 4,971,749 | 891,618 | 24,600,430 |
| AMS42.1 | 40,209,650 | 308 | 9,318,255 | 4,642,451 | 922,866 | 25,533,292 |
| AMS42.2 | 41,472,498 | 296 | 3,913,733 | 6,006,483 | 1,199,154 | 30,464,658 |
| AMS42.3 | 37,960,446 | 301 | 3,962,949 | 5,395,522 | 1,065,873 | 27,658,823 |

| **Pathways gene-ontology term (NMS42 vs NMS37)** | **p-values** |
| --- | --- |
| NADH to cytochrome oxidase electron transfer I | 1.51E-04 |
| NADH to cytochrome oxidase electron transfer I | 2.93E-04 |
| NADH to cytochrome oxidase electron transfer II | 0.001658 |
| NADH to cytochrome oxidase electron transfer II | 0.003303 |
| Superpathway of tetrahydrofolate biosynthesis | 0.006492 |
| Electron Transfer Chains | 0.027331 |
| Aerobic Respiration | 0.027331 |
| Fatty Acid and Lipid Biosynthesis | 0.028097 |
| Single Carbon Carrier Biosynthesis | 0.030802 |
| Folate Biosynthesis | 0.030802 |
| Proteinogenic Amino Acid Biosynthesis | 0.034632 |
| tetrahydrofolate biosynthesis | 0.038211 |
| Respiration | 0.041452 |
| Amino Acid Biosynthesis | 0.043503 |

**Supplementary Table S4.** The enriched Gene ontology (GO) terms (*p*-value ≤ 0.05) of up and down regulated DEGs in *M. capsulatus* Bath cells grown on NMS42 vs NMS 37 represent the GO terms belonging to pathways involved.

**Supplementary Table S5.** The enriched Gene ontology (GO) terms (*p*-value ≤ 0.05) of up and down regulated DEGs in *M. capsulatus* Bath cells grown on AMS42 vs NMS 37 represent the GO terms belonging to pathways involved.

| **Pathways Gene-Ontology-Terms (AMS37 vs NMS37)** | ***p*-values** |
| --- | --- |
| NADH to cytochrome oxidase electron transfer I | 1.61E-07 |
| NADH to cytochrome oxidase electron transfer I | 8.35E-07 |
| Respiration | 2.33E-05 |
| NADH dehydrogenase activity | 3.45E-05 |
| Electron Transfer Chains | 4.06E-05 |
| Aerobic Respiration | 4.06E-05 |
| NADH dehydrogenase (quinone) activity | 1.50E-04 |
| NAD(P)H dehydrogenase (quinone) activity | 2.37E-04 |
| aerobic respiration I (cytochrome c) | 2.51E-04 |
| oxidoreductase activity, acting on NAD(P)H | 3.51E-04 |
| NADH to cytochrome oxidase electron transfer II | 3.85E-04 |
| NADH dehydrogenase (ubiquinone) activity | 3.97E-04 |
| oxidoreductase activity, acting on NAD(P)H, quinone or similar compound as acceptor | 4.41E-04 |
| quinone binding | 5.75E-04 |
| Generation of Precursor Metabolites and Energy | 7.68E-04 |
| oxidoreductase activity | 9.23E-04 |
| nitrate assimilation | 0.001455 |
| NADH to cytochrome oxidase electron transfer II | 0.001753 |
| ferredoxin hydrogenase activity | 0.002846 |
| oxidoreductase activity, acting on hydrogen as donor, iron-sulfur protein as acceptor | 0.002846 |
| oxoacid metabolic process | 0.004247 |
| organic acid metabolic process | 0.004603 |
| ATP synthesis coupled electron transport | 0.004739 |
| oxidoreductase activity, acting on hydrogen as donor | 0.004739 |
| glutamine family amino acid metabolic process | 0.00523 |
| carbonate dehydratase activity | 0.005328 |
| reactive nitrogen species metabolic process | 0.005328 |
| nitrate metabolic process | 0.005328 |
| Proteinogenic Amino Acid Biosynthesis | 0.007816 |
| Nitrogen Compound Metabolism | 0.008055 |
| oxidoreduction-driven active transmembrane transporter activity | 0.008055 |
| respiratory electron transport chain | 0.008565 |
| Amino Acid Biosynthesis | 0.011322 |
| oxidoreductase activity, acting on other nitrogenous compounds as donors | 0.012203 |
| methane monooxygenase complex | 0.012203 |
| pentose phosphate pathway (oxidative branch) I | 0.012203 |
| carbon-oxygen lyase activity | 0.012729 |
| anthranilate synthase activity | 0.012883 |
| nitrite reductase [NAD(P)H] activity | 0.012883 |
| 3-isopropylmalate dehydratase activity | 0.012883 |
| nitrate transport | 0.012883 |
| protein-glutamine N-methyltransferase activity | 0.012883 |
| nitrate transmembrane transporter activity | 0.012883 |
| ammonium transmembrane transporter activity | 0.012883 |
| nitrite reductase activity | 0.012883 |
| glutamate dehydrogenase [NAD(P)+] activity | 0.012883 |
| ammonium transmembrane transport | 0.012883 |
| oxidoreductase activity, acting on the aldehyde or oxo group of donors, disulfide as acceptor | 0.012883 |
| secondary active sulfate transmembrane transporter activity | 0.012883 |
| glutamate dehydrogenase (NAD+) activity | 0.012883 |
| peptidyl-glutamine methylation | 0.012883 |
| hydro-lyase activity | 0.014245 |
| carboxylic acid metabolic process | 0.015436 |
| cation binding | 0.015752 |
| glutamine family amino acid biosynthetic process | 0.019909 |
| metal ion binding | 0.020453 |
| Amino Acid Degradation | 0.022207 |
| 4 iron, 4 sulfur cluster binding | 0.024792 |
| iron-sulfur cluster binding | 0.026881 |
| Pathways | 0.027146 |
| metal cluster binding | 0.029611 |
| catalytic complex | 0.030109 |
| L-proline biosynthesis I (from L-glutamate) | 0.03574 |
| amino acid binding | 0.03574 |
| L-citrulline degradation | 0.03574 |
| Other Amino Acid Degradation | 0.03574 |
| proline metabolic process | 0.03574 |
| oxidoreductase activity, acting on other nitrogenous compounds as donors, with NAD or NADP as acceptor | 0.03574 |
| proline biosynthetic process | 0.03574 |
| glutaminyl-tRNA synthase (glutamine-hydrolyzing) activity | 0.03574 |
| nucleotide-excision repair | 0.03574 |
| L-proline biosynthetic process | 0.03574 |
| L-proline Biosynthesis | 0.03574 |
| Ammonia Oxidation | 0.035902 |
| methane monooxygenase NADPH activity | 0.035902 |
| pentose-phosphate shunt | 0.035902 |
| NADPH regeneration | 0.035902 |
| ammonia oxidation III | 0.035902 |
| methane monooxygenase NADH activity | 0.035902 |
| ammonia oxidation I (aerobic) | 0.035902 |
| inorganic anion transmembrane transporter activity | 0.038624 |
| anion transmembrane transporter activity | 0.038624 |
| cellular amino acid metabolic process | 0.040048 |
| NADP binding | 0.04515 |
| lyase activity | 0.04716 |

**Supplementary Table S6.** The enriched Gene ontology (GO) terms (P-value ≤ 0.05) of up and down regulated DEGs in *M. capsulatus* Bath cells grown on AMS37 vs NMS 37 represent the GO terms belonging to pathways involved

| **Pathways gene-ontology term (AMS42 vs NMS37)** | **p-values** |
| --- | --- |
| NADH to cytochrome oxidase electron transfer I | 2.58E-04 |
| NADH to cytochrome oxidase electron transfer I | 4.57E-04 |
| NADH to cytochrome oxidase electron transfer II | 0.001002354 |
| NADH to cytochrome oxidase electron transfer II | 0.001874605 |
| Electron Transfer Chains | 0.004841733 |
| Aerobic Respiration | 0.004841733 |
| Cell Structure Biosynthesis | 0.008012835 |
| NAD Biosynthesis | 0.008473471 |
| aerobic respiration I (cytochrome c) | 0.008786486 |
| Respiration | 0.010864913 |
| Fatty Acid and Lipid Biosynthesis | 0.01572838 |
| NAD Metabolism | 0.021233138 |
| NAD biosynthesis I (from aspartate) | 0.03338829 |
| Other Amino Acid Biosynthesis | 0.035643846 |
| L-histidine Biosynthesis | 0.035643846 |
| L-histidine biosynthesis | 0.035643846 |
| Peptidoglycan Biosynthesis | 0.042177208 |
| Cell Wall Biosynthesis | 0.042177208 |
| aerobic respiration II (cytochrome c) (yeast) | 0.043247476 |

**Supplementary Table S7.** Transcript abundance of genes involved in oxidative phosphorylation pathway in *M. capsulatus* Bath

| **gene** | **protein_id** | **gene.y** | **product** | **N42 vs N37** | **A37 vs N37** | **A42 vs N37** |
| --- | --- | --- | --- | --- | --- | --- |
| **Complex I** |  |  |  |  |  |  |
| MCA_RS02510 | WP_010959859.1 | *nuoM1* | NADH-quinone oxidoreductase subunit M | -1.92 | -1.02 | -1.33 |
| MCA_RS02525 | WP_010959862.1 | *nuoM2* | NADH-quinone oxidoreductase subunit M | -1.92 | -1.12 | -1.83 |
| MCA_RS02530 | WP_010959863.1 | *nuoM3* | NADH-quinone oxidoreductase subunit M | -2.08 | -1.17 | -1.9 |
| MCA_RS06630 | WP_010960627.1 | *nuoN* | NADH-quinone oxidoreductase subunit N | 8.56 | 4.51 | 6.25 |
| MCA_RS06635 | WP_143708489.1 | *nuoM4* | NADH-quinone oxidoreductase subunit M | 8.71 | 4.55 | 7.69 |
| MCA_RS06640 | WP_010960629.1 | *nuoL* | NADH-quinone oxidoreductase subunit L | 9.32 | 4.73 | 8.41 |
| MCA_RS06645 | WP_010960630.1 | *nuoK* | NADH-quinone oxidoreductase subunit NuoK | 7.73 | 4.4 | 5.84 |
| MCA_RS06650 | WP_010960631.1 | *nuoJ* | NADH-quinone oxidoreductase subunit J | 8.52 | 4.13 | 5.65 |
| MCA_RS06655 | WP_010960632.1 | *nuoI* | NADH-quinone oxidoreductase subunit NuoI | 10.31 | 4.93 | 5.95 |
| MCA_RS06660 | WP_010960633.1 | *nuoH* | NADH-quinone oxidoreductase subunit NuoH | 7.42 | 4.28 | 5.27 |
| MCA_RS06665 | WP_010960634.1 | *nuoG* | NADH-quinone oxidoreductase subunit NuoG | 8.41 | 3.86 | 8.15 |
| MCA_RS06670 | WP_010960635.1 | *nuoF* | NADH-quinone oxidoreductase subunit NuoF | 6.05 | 3.11 | 5.45 |
| MCA_RS06675 | WP_010960636.1 | *nuoE* | NADH-quinone oxidoreductase subunit NuoE | 5.97 | 3.06 | 3.93 |
| MCA_RS06680 | WP_010960637.1 | *nuoCD* | NADH-quinone oxidoreductase subunit C/D | 5.34 | 3.24 | 5.47 |
| MCA_RS06685 | WP_010960638.1 | *nuoB* | NADH-quinone oxidoreductase subunit B | 4.88 | 3.97 | 5.04 |
| MCA_RS06690 | WP_010960639.1 | *nuoA* | NADH-quinone oxidoreductase subunit A | 3.05 | 2.79 | 3.25 |
| MCA_RS09405 | WP_010961166.1 | *ndh* | NAD(P)/FAD-dependent oxidoreductase | 1.67 | 1.43 | 1.65 |
| **Complex II** |  |  |  |  |  |  |
| MCA_RS07600 | WP_010960814.1 | *sdhB* | succinate dehydrogenase iron-sulfur subunit | -1.32 | -1.17 | -1.65 |
| MCA_RS07635 | WP_010960820.1 | *sdhC* | succinate dehydrogenase, cytochrome b556 subunit | -1.22 | -1.20 | -1.16 |
| MCA_RS07640 | WP_010960821.1 | *sdhD* | succinate dehydrogenase, hydrophobic membrane anchor protein | -1.31 | -1.54 | -1.31 |
| MCA_RS07645 | WP_010960822.1 | *sdhA* | succinate dehydrogenase flavoprotein subunit | -1.84 | -1.53 | -1.97 |
| **Complex III** |  |  |  |  |  |  |
| MCA_RS09600 | WP_010961206.1 | *petC* | cytochrome c1 | -1.34 | -1.07 | -1.66 |
| MCA_RS09605 | WP_010961207.1 | *petB* | cytochrome bc complex cytochrome b subunit | -1.56 | -1.14 | -1.43 |
| MCA_RS09610 | WP_010961208.1 | *petA* | ubiquinol-cytochrome c reductase iron-sulfur subunit | -1.15 | -1.08 | -1.04 |
| **Complex IV** |  |  |  |  |  |  |
| MCA_RS04330 | WP_010960199.1 | *coxB1* | cytochrome c oxidase subunit II | -1.16 | 1.09 | 1.05 |
| MCA_RS04335 | WP_010960200.1 | *coxA1* | cytochrome c oxidase subunit I | -1.30 | -1.05 | -1.30 |
| MCA_RS04345 | WP_010960203.1 | *coxC* | cytochrome c oxidase subunit 3 | -1.55 | -1.11 | -1.94 |
| MCA_RS11755 | WP_010961622.1 | *coxA2* | b(o/a)3-type cytochrome-c oxidase subunit 1 | 1.36 | 1.22 | 1.80 |
| MCA_RS11760 | WP_143708613.1 | *coxB2* | cytochrome C oxidase subunit II | 1.08 | 1.05 | 1.53 |
| **Complex V** |  |  |  |  |  |  |
| MCA_RS00030 | WP_010959379.1 | *atp-B1* | F0F1 ATP synthase subunit A | -1.41 | -1.21 | -1.31 |
| MCA_RS00035 | WP_010959380.1 | *atp-E1* | F0F1 ATP synthase subunit C | -2.37 | -1.78 | -2.47 |
| MCA_RS00040 | WP_010959381.1 | *atp-F* | F0F1 ATP synthase subunit B | -2.45 | -1.94 | -2.83 |
| MCA_RS00045 | WP_010959382.1 | *atp-H* | F0F1 ATP synthase subunit delta | -2.94 | -1.91 | -3.22 |
| MCA_RS00050 | WP_010959383.1 | *atp-A1* | F0F1 ATP synthase subunit alpha | -2.83 | -1.83 | -3.12 |
| MCA_RS00055 | WP_010959384.1 | *atpG-1* | F0F1 ATP synthase subunit gamma | -3.34 | -1.87 | -4.00 |
| MCA_RS00060 | WP_010959385.1 | *atpD-1* | F0F1 ATP synthase subunit beta | -3.16 | -1.74 | -3.58 |
| MCA_RS00065 | WP_010959386.1 | *atpC-1* | ATP synthase epsilon chain 1 | -3.26 | -1.45 | -2.86 |
| MCA_RS07670 | WP_017365699.1 | *atpD* | F0F1 ATP synthase subunit beta | 1.08 | -1.11 | 1.12 |
| MCA_RS07675 | WP_010960829.1 | MCA_RS07675 | putative ATP synthase F1 | -1.10 | -1.13 | -1.18 |
| MCA_RS13180 | WP_010961902.1 | *atpB-2* | single-stranded DNA-binding protein | -1.35 | -1.18 | -1.66 |
| MCA_RS13215 | WP_010961911.1 | *atpE-2* | ATP synthase F0 subunit C | 1.19 | -1.13 | 1.25 |
| MCA_RS13220 | WP_010961912.1 | MCA_RS13220 | ATP synthase subunit b 2 | 1.01 | -1.18 | -1.12 |
| MCA_RS13245 | WP_010961916.1 | *atpA-2* | F0F1 ATP synthase subunit alpha | 1.56 | 1.40 | 1.96 |
| MCA_RS13250 | WP_010961917.1 | *atpG-2* | F0F1 ATP synthase subunit gamma | 1.35 | 1.23 | 1.22 |
| MCA_RS14820 | WP_010962209.1 | *atpC-2* | F0F1 ATP synthase subunit epsilon | -1.52 | -1.04 | -1.34 |

**Supplementary Table S8.** Predicted genes of methane oxidation pathway in *M. capsulatus* Bath

| **gene** | **protein_id** | **gene.y** | **product** | **N42 vs N37** | **A37 vs N37** | **A42 vs N37** |
| --- | --- | --- | --- | --- | --- | --- |
| **Methane to methanol** | | | | | | |
| MCA_RS05885 | WP_010960482.1 | mmoX | methane monooxygenase component A alpha chain | 2.61 | 1.74 | 7.38 |
| MCA_RS05890 | WP_010960483.1 | mmoY | methane monooxygenase component A subunit beta | 1.54 | 1.47 | 3.04 |
| MCA_RS05895 | WP_010960484.1 | mmoB | methane monooxygenase regulatory protein B | 1.78 | 1.53 | 2.54 |
| MCA_RS05905 | WP_010960485.1 | mmoZ | methane monooxygenase component A subunit gamma | 2.10 | 1.70 | 3.07 |
| MCA_RS05910 | WP_010960486.1 | mmoD | soluble methane monooxygenase-binding protein MmoD | 1.40 | 1.10 | 1.60 |
| MCA_RS01450 | WP_010959659.1 | *pmoC3* | methane monooxygenase/ammonia monooxygenase subunit C | 1.42 | 2.03 | 1.25 |
| MCA_RS08830 | WP_010961049.1 | *pmoB1* | methane monooxygenase/ammonia monooxygenase subunit B | -1.08 | 1.46 | -1.29 |
| MCA_RS08835 | WP_010961050.1 | *pmoA1* | methane monooxygenase/ammonia monooxygenase subunit A | 1.05 | 1.76 | -1.01 |
| MCA_RS08840 | WP_010961051.1 | *pmoC1* | methane monooxygenase/ammonia monooxygenase subunit C | 1.24 | 1.92 | 1.26 |
| MCA_RS13975 | WP_010961050.1 | *pmoA2* | methane monooxygenase/ammonia monooxygenase subunit A | 1.05 | 1.80 | -1.01 |
| MCA_RS13980 | WP_010961051.1 | *pmoC2* | methane monooxygenase/ammonia monooxygenase subunit C | 1.22 | 1.98 | 1.23 |
| MCA_RS13970 | WP_010961049.1 | *pmoB2* | methane monooxygenase/ammonia monooxygenase subunit B | -1.12 | 1.50 | -1.31 |
| **Methanol to formaldehyde** | | | | | | |
| MCA_RS01470 | WP_010959663.1 | PQQ-mdh | methanol/ethanol family PQQ-dependent dehydrogenase | 3.06 | 2.57 | 10.23 |
| MCA_RS03850 | WP_010960106.1 | mxaF | methanol/ethanol family PQQ-dependent dehydrogenase | -1.93 | 1.19 | -3.67 |
| **Formaldehyde to formate** | | | | | | |
| MCA_RS14030 | WP_010962061.1 | fae | formaldehyde-activating enzyme | -3.76 | -2.26 | -3.44 |
| MCA_RS14015 | WP_010962058.1 | mch | methenyltetrahydromethanopterin cyclohydrolase | -1.46 | -1.07 | -1.75 |
| MCA_RS11360 | WP_010961547.1 | MCA_RS11360 | formylmethanofuran dehydrogenase subunit A | -2.78 | -1.67 | -2.12 |
| MCA_RS13985 | WP_010962052.1 | MCA_RS13985 | formylmethanofuran dehydrogenase subunit C | -2.12 | -1.18 | -2.37 |
| MCA_RS13990 | WP_010962053.1 | fhcD | formylmethanofuran--tetrahydromethanopterin N-formyltransferase | -2.27 | -1.24 | -2.44 |
| MCA_RS13995 | WP_010962054.1 | MCA_RS13995 | formylmethanofuran dehydrogenase subunit A | -2.10 | -1.28 | -1.70 |
| MCA_RS14000 | WP_010962055.1 | MCA_RS14000 | formylmethanofuran dehydrogenase subunit B | -1.86 | 1.05 | -1.16 |
| MCA_RS06845 | WP_010960668.1 | MCA_RS06845 | formate dehydrogenase subunit alpha | -1.09 | -1.39 | -1.98 |
| MCA_RS00685 | WP_041360546.1 | metF | methylenetetrahydrofolate reductase [NAD(P)H] | -3.63 | -2.17 | -4.34 |
| MCA_RS02495 | WP_010959856.1 | MCA_RS02495 | methylenetetrahydrofolate dehydrogenase | 1.07 | 1.22 | 1.54 |
| MCA_RS13570 | WP_041361342.1 | MCA_RS13570 | 5-formyltetrahydrofolate cyclo-ligase | 1.40 | -1.03 | 1.63 |
| MCA_RS14805 | WP_010962206.1 | MCA_RS14805 | methylenetetrahydromethanopterin dehydrogenase | -1.87 | -1.20 | -1.42 |
| MCA_RS14810 | WP_041361389.1 | MCA_RS14810 | methylenetetrahydromethanopterin dehydrogenase | -1.25 | 1.07 | 1.13 |
| MCA_RS02490 | WP_010959855.1 | MCA_RS02490 | cyclodeaminase/cyclohydrolase family protein | -1.04 | 1.22 | 1.33 |

| **Formate to CO_2_** | | | | | | |
| --- | --- | --- | --- | --- | --- | --- |
| MCA_RS05955 | WP_010960494.1 | MCA_RS05955 | formate dehydrogenase subunit gamma | 1.28 | 1.10 | -1.99 |
| MCA_RS05960 | WP_010960495.1 | *fdxH* | formate dehydrogenase subunit beta | 1.24 | 1.01 | -1.75 |
| MCA_RS05965 | WP_010960496.1 | *fdnG* | formate dehydrogenase-N subunit alpha | 1.26 | 1.21 | -1.11 |
| MCA_RS06835 | WP_010960666.1 | MCA_RS06835 | formate dehydrogenase subunit delta | -1.02 | -1.30 | -2.34 |
| MCA_RS06845 | WP_010960668.1 | MCA_RS06845 | formate dehydrogenase subunit alpha | -1.09 | -1.39 | -1.98 |
| MCA_RS06855 | WP_010960670.1 | MCA_RS06855 | formate dehydrogenase subunit gamma | 1.41 | -1.10 | 1.14 |
| MCA_RS12620 | WP_010961792.1 | MCA_RS12620 | formate dehydrogenase subunit alpha | -1.30 | 1.46 | -2.03 |

**Supp. Table S9.** Predicted genes of nitrogen pathway in *M. capsulatus* Bath

| **gene** | **protein_id** | **gene.y** | **product** | **N42vsN37** | **A37vsN37** | **A42vsN37** |
| --- | --- | --- | --- | --- | --- | --- |
| MCA_RS01320 | WP_010959632.1 | *amt-1* | ammonium transporter | 2.64 | 3.29 | 5.71 |
| MCA_RS02425 | WP_143708442.1 | *amt-2* | ammonium transporter | 6.26 | 14.49 | 17.07 |
| MCA_RS07770 | WP_017365298.1 | *amt-4* | ammonium transporter | 1.70 | -6.90 | -1.75 |
| MCA_RS10445 | WP_010961375.1 | *amt-3* | ammonium transporter | 1.36 | -3.27 | -1.83 |
| MCA_RS02950 | WP_010959939.1 | *MCA_RS02950* | NarK/NasA family nitrate transporter | 1.24 | -74.25 | -19.76 |
| MCA_RS02930 | WP_010959935.1 | *nasA* | nitrate reductase | -2.13 | -12.53 | -13.41 |
| MCA_RS02935 | WP_010959936.1 | *nirD* | nitrite reductase small subunit NirD | -2.10 | -12.51 | -10.04 |
| MCA_RS11770 | WP_010961626.1 | *MCA_RS11770* | nitric oxide reductase | -1.85 | -1.36 | -1.19 |
| MCA_RS04695 | WP_081423403.1 | *hao* | hydroxylamine reductase | 1.16 | 2.46 | 1.98 |
| MCA_RS01450 | WP_010959659.1 | *pmoC3* | methane monooxygenase/ammonia monooxygenase subunit C | 1.42 | 2.03 | 1.25 |
| MCA_RS08830 | WP_010961049.1 | *pmoB1* | methane monooxygenase/ammonia monooxygenase subunit B | -1.08 | 1.46 | 2.29 |
| MCA_RS08835 | WP_010961050.1 | *pmoA1* | methane monooxygenase/ammonia monooxygenase subunit A | 1.05 | 1.76 | 2.01 |
| MCA_RS08840 | WP_010961051.1 | *pmoC1* | methane monooxygenase/ammonia monooxygenase subunit C | 1.24 | 1.92 | 1.26 |
| MCA_RS13970 | WP_010961049.1 | *pmoB2* | methane monooxygenase/ammonia monooxygenase subunit B | -1.12 | 1.50 | -1.31 |
| MCA_RS13975 | WP_010961050.1 | *pmoA2* | methane monooxygenase/ammonia monooxygenase subunit A | 1.05 | 1.80 | -1.01 |
| MCA_RS13980 | WP_010961051.1 | *pmoC2* | methane monooxygenase/ammonia monooxygenase subunit C | 1.22 | 1.98 | 2.23 |
| MCA_RS05050 | WP_010960331.1 | *gluD* | Glu/Leu/Phe/Val dehydrogenase | -1.49 | -2.26 | -1.19 |
| MCA_RS09975 | WP_010961284.1 | *gltB* | glutamate synthase large subunit | -2.55 | -1.42 | -1.74 |
| MCA_RS09980 | WP_010961285.1 | *gltD* | glutamate synthase subunit beta | -3.03 | -1.51 | -3.18 |
| MCA_RS08255 | WP_010960935.1 | *glnA* | glutamate--ammonia ligase | -1.52 | -1.48 | -1.68 |
| MCA_RS02795 | WP_010959911.1 | *ald* | alanine dehydrogenase | -1.34 | 1.85 | 1.94 |
